# Foliar Pathogen Infection Manipulates Soil Health through Root Exudate-Modified Rhizosphere Microbiome

**DOI:** 10.1128/spectrum.02418-22

**Published:** 2022-11-29

**Authors:** Lifen Luo, Junxing Zhang, Chen Ye, Su Li, Shengshuang Duan, Zhengping Wang, Huichuan Huang, Yixiang Liu, Weiping Deng, Xinyue Mei, Xiahong He, Min Yang, Shusheng Zhu

**Affiliations:** a State Key Laboratory for Conservation and Utilization of Bio-Resources in Yunnan, Yunnan Agricultural University, Kunming, China; b Key Laboratory for Agro-biodiversity and Pest Control of Ministry of Education, College of Plant Protection, Yunnan Agricultural University, Kunming, China; c National Engineering Research Center for Applied Technology of Agricultural Biodiversity, College of Plant Protection, Yunnan Agricultural University, Kunming, China; Pennsylvania State University

**Keywords:** plant-soil feedback, rhizosphere microbiome, *Ilyonectria destructans*, soilborne disease, *Alternaria panax*, plant-soil interaction, root exudates

## Abstract

Negative plant-soil feedback (NPSF) due to the buildup of soilborne pathogens in soil is a major obstacle in sustainable agricultural systems. Beneficial rhizosphere microfloras are recruited by plants, and mediating this has become a strategic priority to manipulate plant health. Here, we found that foliar infection of Panax notoginseng by Alternaria panax changed plant-soil feedback from negative to positive. Foliar infection modified the rhizosphere soil microbial community and reversed the direction of the buildup of the soilborne pathogen Ilyonectria destructans and beneficial microbes, including *Trichoderma*, *Bacillus*, and *Streptomyces*, in rhizosphere soil. These beneficial microbes not only showed antagonistic ability against the pathogen *I. destructans* but also enhanced the resistance of plants to *A. panax*. Foliar infection enhanced the exudation of short- and long-chain organic acids, sugars, and amino acids from roots. *In vitro* and *in vivo* experiments validated that short- and long-chain organic acids and sugars play dual roles in simultaneously suppressing pathogens but enriching beneficial microbes. In summary, foliar infection could change root secretion to drive shifts in the rhizosphere microbial community to enhance soil health, providing a new strategy to alleviate belowground disease in plants through aboveground inducement.

**IMPORTANCE** Belowground soilborne disease is the main factor limiting sustainable agricultural production and is difficult to manage due to the complexity of the soil environment. Here, we found that aboveground parts of plants infected by foliar pathogens could enhance the secretion of organic acids, sugars, and amino acids in root exudates to suppress soilborne pathogens and enrich beneficial microbes, eventually changing the plant and soil feedback from negative to positive and alleviating belowground soilborne disease. This is an exciting strategy by which to achieve belowground soilborne disease management by manipulating the aboveground state through aboveground stimulation.

## INTRODUCTION

Plant-soil feedback (PSF) is the phenomenon by which plants affect the soil, which in turn affects the growth of the same or other plants (1). Root-associated microorganisms affect plant or plant offspring performance either negatively or positively, eventually determining the direction and strength of PSF (positive, negative, or neutral feedback) (2, 3). Positive plant-soil feedbacks (PPSFs) have been manifested in many ways, such as a positive legacy for successive crops and increased productivity through belowground facilitation, which is key to improving the sustainability of food production while maintaining productivity (3, 4). Negative plant-soil feedbacks (NPSFs) make soil less suitable for the same or other individuals of the same species (2) and play important roles in maintaining plant diversity and driving community dynamics in natural ecosystems (5). However, NPSF is known as an important factor limiting crop productivity in intensive agricultural systems (6).

NPSF is caused by abiotic and biotic factors (7, 8). The changes in rhizospheric microbes, especially the build-up of soilborne pathogens, have been proven to be the major driving factors of NPSF (5, 9). Many studies have found that NPSF could be alleviated when specific rhizosphere soilborne pathogens were suppressed or the abundance of beneficial symbionts was enriched (10, 11). Therefore, many strategies, including crop rotation, soil sterilization with chemical and physical methods, exogenous application of plant growth-promoting rhizobacteria (PGPRs), and so on, have been employed to alleviate NPSF in agricultural production systems (12–15). However, these measures all face limits. Among them, soil chemical and physical sterilization approaches often pose environmental risks or allow for recolonization by soilborne pathogens (15, 16). Biological control in fields by exogenous application of PGPRs is not often stable because of the complexity of the soil environment (17, 18). Thus, a new environmentally friendly strategy to alleviate NPSF by stimulating the capability of the rhizosphere microbiome to suppress soilborne pathogens should be developed.

Plants can employ different strategies to resist biotic and abiotic stresses. Apart from internal immunological strategies that involve physiological and genetic modifications at the cellular level, plants could employ external strategies that rely on the recruitment of beneficial organisms (19). A series of studies demonstrated that plants under the stress of long-term soilborne pathogen infection could enrich antagonistic microflora to form disease-suppressive soil to reduce the severity of soilborne disease (20–23). When Arabidopsis thaliana was challenged with foliar pathogens (such as Pseudomonas syringae pv. tomato and Hyaloperonospora arabidopsidis) or wounds made by insects, the roots recruited beneficial microbes to help themselves against infection by foliar pathogens (24–27). Belowground soilborne diseases are difficult to manage by direct soil modification due to the complexity of the soil environment. Therefore, the recruitment of beneficial rhizosphere microbiomes to alleviate underground soilborne diseases of plants through aboveground inducement is a desirable approach. Manipulation of the exudate composition from root apices shapes the microbiome throughout the root system (28). A core idea of acupuncture to treat human diseases is that stimulation at specific body regions (acupoints) can modulate body physiology at distant sites by activating this particular signaling pathway (29). Thus, whether aboveground stimulation could induce changes in plant metabolism and root secretion to recruit beneficial microbes to suppress belowground soilborne pathogens and then alleviate NPSF deserves further study.

The *Panax* genus, belonging to the Araliaceae, contains commonly used medicinal crops that can modulate blood pressure, improve metabolism, and strengthen the immune system (15, 30). However, these crops exhibit strong NPSF and are subject to serious soilborne disease in the field (31, 32). In this study, we used P. notoginseng, one of the species in the *Panax* genus that experiences a strong NPSF (15, 32, 33), as a model crop to study (i) whether the aboveground infection by the foliar pathogen Alternaria panax could alleviate NPSF caused by the soilborne pathogen, (ii) the function of the rhizosphere microbiome in soilborne disease suppression and its dynamic changes when the plant is infected by *A. panax*, and (iii) the change in root exudates after foliar infection and then decipher the function of infection-induced root exudates in modifying the rhizosphere microbiota. These studies will help us to further understand the mechanism by which biotic stress redirects NPSF and develop a novel technique to manipulate the rhizosphere microbiome to alleviate soilborne disease.

## RESULTS

### Foliar infection changed the plant-soil feedback from negative to positive.

**FIG 1:**
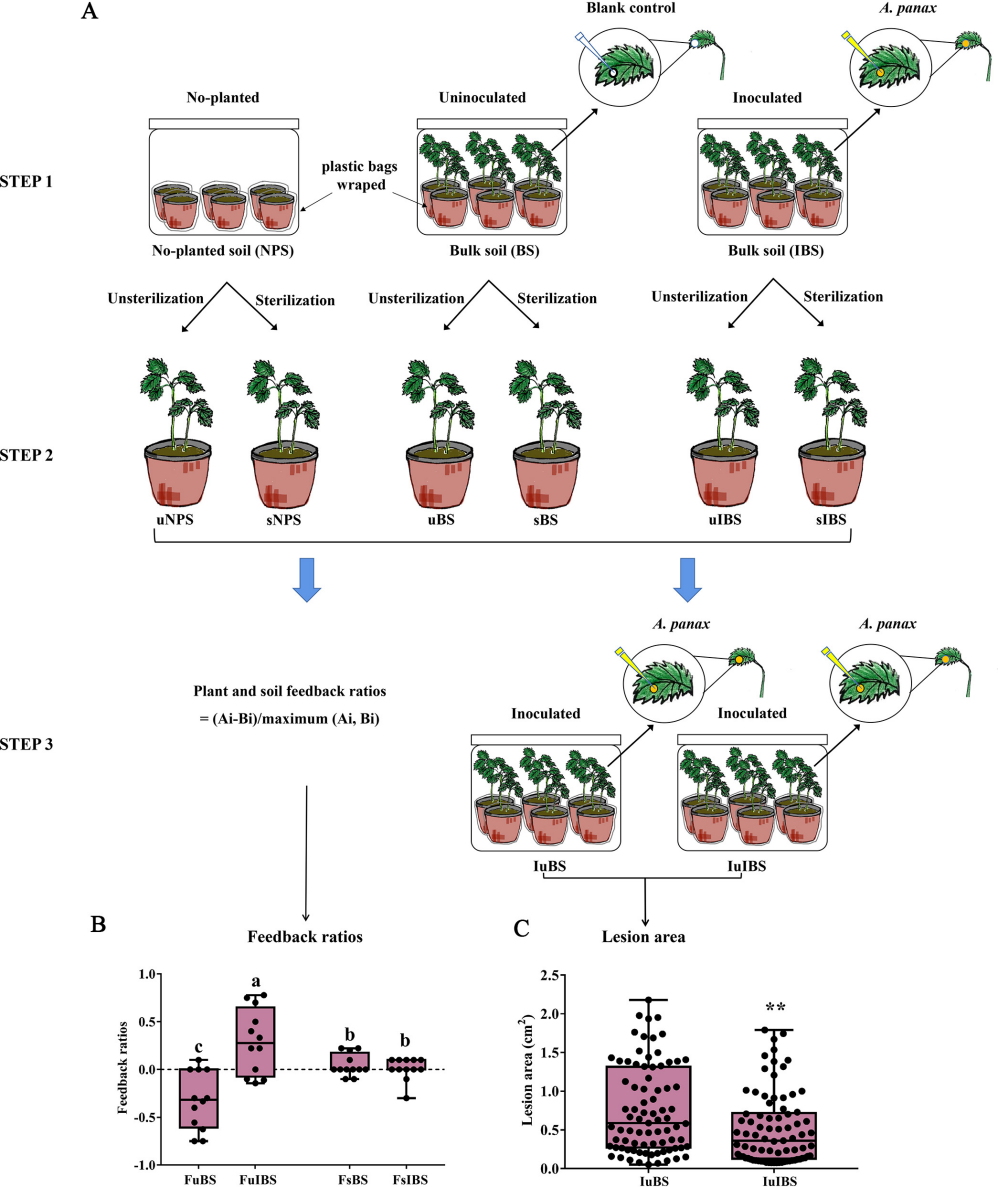
Effect of foliar infection on plant-soil feedback (PSF) and foliar disease resistance. (A) The experimental process used to assess the effect of foliar infection on the plant-soil feedback and foliar resistance of the succeeding generation. In step 1, three treatments, no-plant soil and *P. notoginseng* with and without foliar inoculation, were applied to soils collected from *Pinus yunnanensis*, *P. kesiya*, and *Eucalyptus* forests. In step 2, the bulk soils from the three treatments were divided equally into two parts. One part of the soil was sterilized at 121°C for 20 min, and the other was not treated. Sterilized seeds were planted, and the seed emergence rate was recorded. Feedback ratios were calculated for each replicate pair as (Ai-Bi)/maximum (Ai, Bi), where Ai and Bi represent the emergence rate in bulk soil (Ai) with or without inoculation and corresponding control (Bi). In step 3, leaves of *P. notoginseng* grown on inoculated- and uninoculated bulk soils without sterilization were inoculated with *A. panax.* (B and C) The box figure shows feedback ratios (B) and lesion areas (C). FuBS and FsBS represent the feedback ratios in nonsterilized or sterilized bulk soils, respectively, based on emergence rates. FuIBS and FsIBS represent the feedback ratios in nonsterilized or sterilized bulk soils, respectively, after foliar infection treatment. IuBS and IuIBS represent the lesion area in bulk soils without or with foliar infection, respectively. Different letters on the bars indicate significant differences between different treatments (*P < *0.05). **, *P* < 0.01.

The seeds showed a significantly higher emergence rate in the bulk soil of *P. notoginseng* with foliar infection by *A. panax* (85.8%) than that without foliar infection (59.2%). After sterilization, the emergence rates in the two treated soils reached more than 90% (see Fig. S1 in the supplemental material). The plant-soil feedback ratio from the bulk soil of *P. notoginseng* was negative (feedback ratios of <0) (Fig. 1B). However, the plant-soil feedback ratio from the bulk soil of *P. notoginseng* with foliar infection by *A. panax* was changed to positive (feedback ratios of >0) (Fig. 1B). Interestingly, this shift disappeared after soil sterilization at 121°C for 20 min (Fig. 1B). Furthermore, *P. notoginseng* seedlings growing in the bulk soil of *P. notoginseng* with foliar infection showed stronger resistance against *A. panax* infection than plants without foliar infection according to the lesion areas (Fig. 1C).

### Foliar infection reversed the direction of soilborne pathogen and beneficial microbe buildup in the rhizosphere.

**FIG 2:**
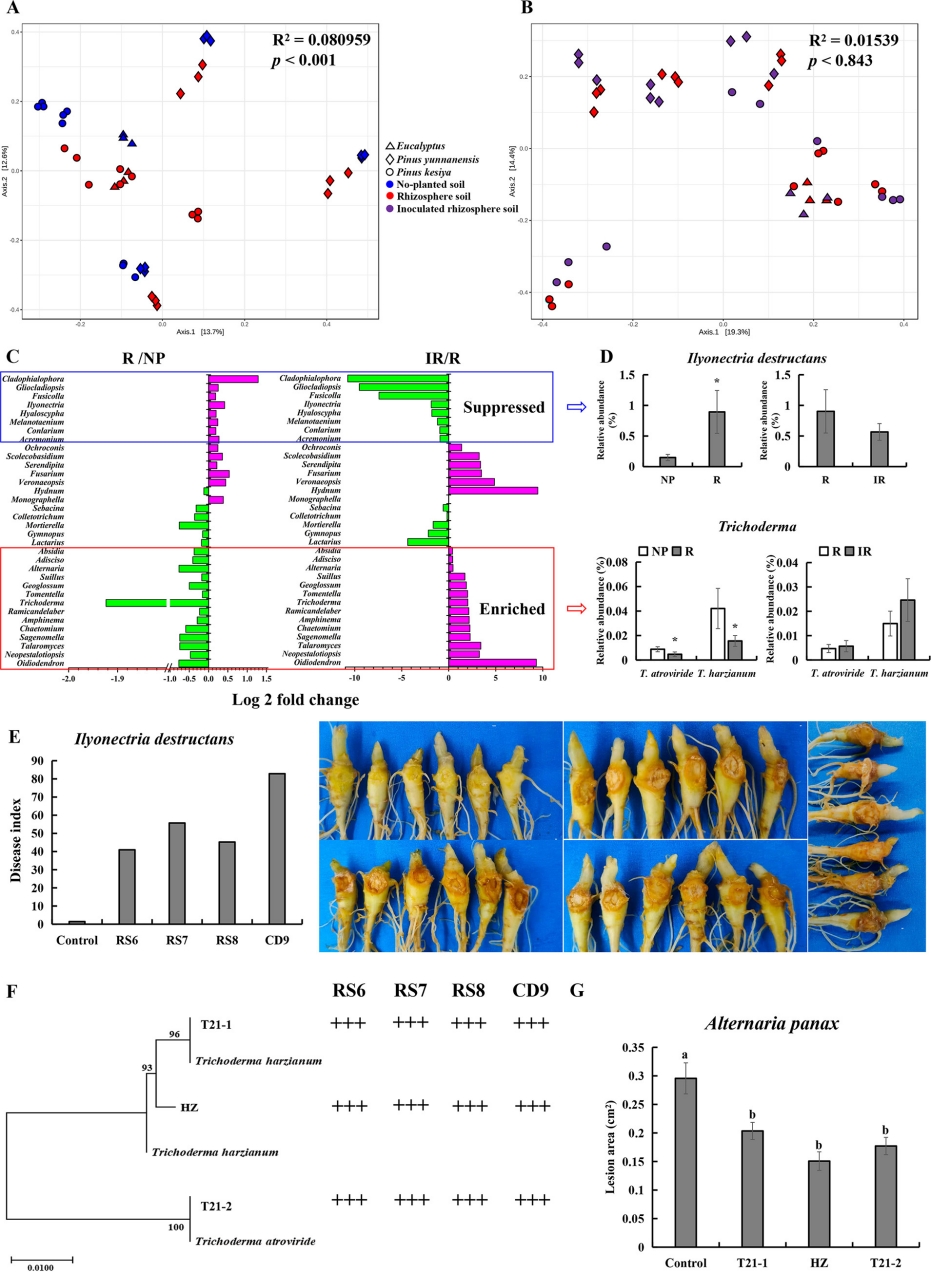
Foliar infection suppressed soilborne pathogens but enriched beneficial fungi. (A and B) PCoA of the fungal community in uninoculated plant rhizospheres and corresponding no-plant (A) or inoculated plant rhizosphere soils (B) based on the Bray distance metric. (C) Comparison of fungal abundance differences in no-plant, uninoculated and inoculated *P. notoginseng* rhizosphere soils at the genus level. “Suppressed” means the abundance of the genus was significantly upregulated in rhizosphere soil without foliar inoculation but downregulated in rhizosphere soil with foliar inoculation compared with the corresponding control. “Enriched” means the abundance of the genus was significantly downregulated in rhizosphere soil without foliar inoculation but upregulated in rhizosphere soil with foliar inoculation compared with the corresponding control. (D) Comparison of characteristic fungal abundance differences in no-plant (NP), uninoculated (R), and inoculated rhizosphere (IR) soils at the species level. *, *P <* 0.05. (E) Pathogenicity tests of *I. destructans* on roots *in vitro*. (F) Hierarchical clustering of ITS genes of marker fungi and their antagonistic activity against *I. destructans.* +++, antimicrobial rate of >60%. (G) Differences in lesion areas on leaves after inoculation with beneficial fungi in the rhizosphere soil. The values represent the means ± standard errors (SEs). Different letters on the bars indicate significant differences between different treatments (*P < *0.05).

**FIG 3:**
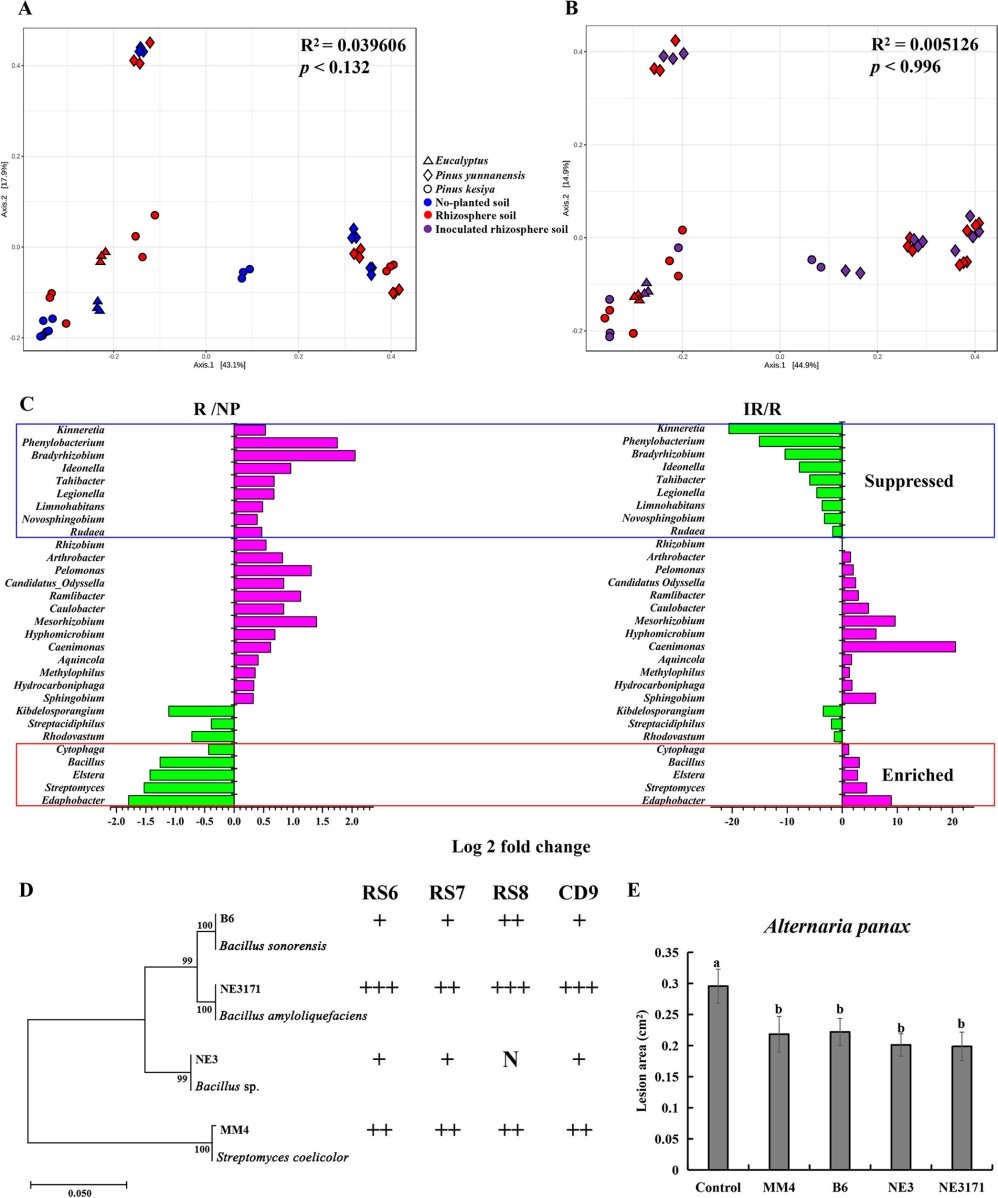
Foliar infection enriched beneficial bacteria. (A and B) PCoA of the bacterial community in the uninoculated plant rhizosphere and corresponding no-plant (A) or inoculated plant rhizosphere soils (B) based on the Bray distance metric. (C) Comparison of characteristic bacterial abundance differences in no-plant (NP), uninoculated (R), and inoculated (IR) rhizosphere soils at the genus level. “Suppressed means the abundance of the genus was significantly upregulated in rhizosphere soil without foliar inoculation but downregulated in rhizosphere soil with foliar inoculation compared with the corresponding control. “Enriched” means the abundance of the genus was significantly downregulated in rhizosphere soil without foliar inoculation but upregulated in rhizosphere soil with foliar inoculation compared with the corresponding control. (D) Hierarchical clustering of 16S rDNA genes of marker bacteria and their antagonistic activity against *I. destructans*. N, +, ++, and +++ represent the levels of antagonistic activity of the strains against *I. destructans*. N, antimicrobial rate of <0; +, antimicrobial rate of 0 to 30%; ++, antimicrobial rate of 30 to 60%; +++, antimicrobial rate of >60%. (E) Differences in lesion areas on leaves after inoculation with beneficial bacteria in rhizosphere soil. The values represent the means ± SEs. Different letters on the bars indicate significant differences between different treatments (*P < *0.05).

We analyzed the fungal and bacterial communities in no-plant soil as well as the rhizosphere soil of *P. notoginseng* with or without foliar infection by *A. panax* (Table S3). Principal-coordinate analysis (PCoA) showed that *P. notoginseng* drove clear shifts in the rhizospheric fungal communities but not the rhizospheric bacterial communities, regardless of whether the soil originated from Pinus yunnanensis, P. kesiya, or *Eucalyptus* forests (Fig. 2A and 3A). At the phylum level, *P. notoginseng* significantly enriched the relative abundance of fungal *Ascomycota* (*P < *0.01) and bacterial *Proteobacteria* (*P < *0.01) but suppressed the abundance of fungal *Basidiomycota* and *Zygomycota* (*P < *0.01) as well as bacterial *Planctomycetes* and *Cyanobacteria* (*P < *0.05) in the rhizosphere soil compared with the no-plant soil (Fig. S2A and C). There were no significant shifts for fungi and bacteria at the community (Fig. 2B and 3B) and phylum (Fig. S2B and D) levels in the rhizosphere soil with or without foliar infection.

Further analysis showed that 34 fungal genera in no-plant soil and rhizosphere soils with or without foliar infection were significantly modified; 14 genera were significantly enriched, but 20 genera were significantly suppressed by plants compared with no-plant soil (Fig. 2C). Among them, 8 *P. notoginseng*-enriched genera and 14 *P. notoginseng*-suppressed genera showed completely opposite trends in the rhizosphere when the plant leaves were infected by *A. panax* (Fig. 2C).

Importantly, the relative abundance of Ilyonectria destructans, which was identified as a root rot pathogen, was significantly enriched in the rhizosphere soil but suppressed in the rhizosphere soil of *P. notoginseng* with foliar infection (Fig. 2D and E). Trichoderma harzianum and Trichoderma atroviride, isolated from rhizosphere soil and identified as beneficial microbes, were significantly suppressed in the rhizosphere soil but enriched in the rhizosphere soil of *P. notoginseng* after infection by *A. panax* (Fig. 2D, F, and G).

With respect to bacteria, nine *P. notoginseng*-enriched genera and five *P. notoginseng*-suppressed genera in rhizosphere soil changed in the opposite direction after foliar infection (Fig. 3C). Similarly, *Bacillus* and *Streptomyces*, isolated from soil and identified as beneficial microbes were significantly suppressed in the rhizosphere soil but enriched in the rhizosphere soil of *P. notoginseng* after infection with *A. panax* (Fig. 3C to E).

### Foliar infection altered the metabolite profiles in plants and root exudates.

**FIG 4:**
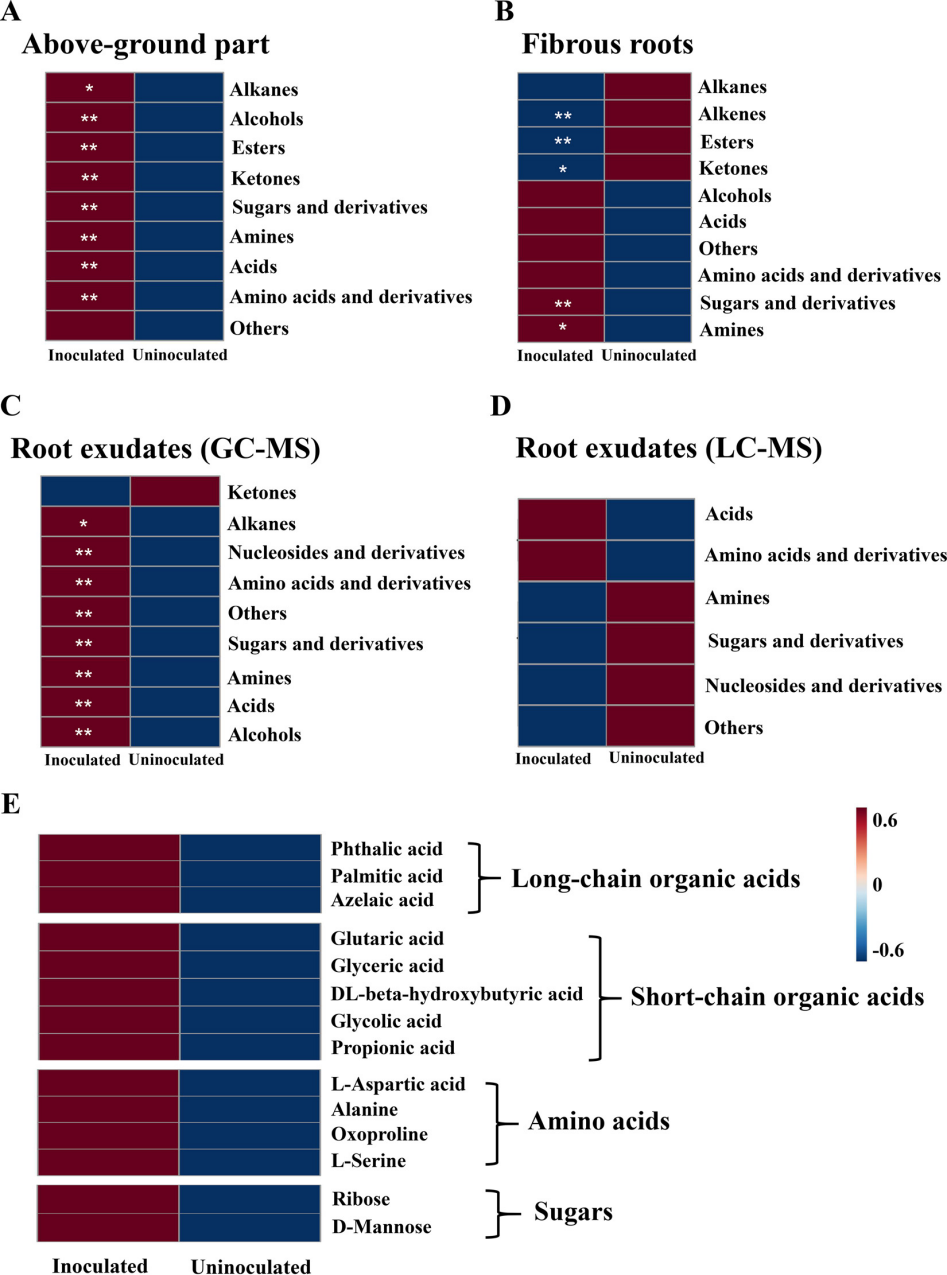
Foliar infection changes the metabolite profiles of aboveground plant parts (leaves and stems), belowground parts (fibrous roots), and root exudates. (A to C) Abundance (cumulative peak height) of compound categories in aboveground parts (A), fibrous roots (B), and root exudates (C) determined by GC-MS analyses. (D) Abundance (cumulative peak height) of compound categories in root exudates determined by UPLC-QTOF-MS/MS analyses. (E) Heatmap analysis of foliar infection-modified metabolites in root exudates by comprehensive GC-MS and UHPLC-QTOF-MS/MS analyses. *, *P <* 0.05; **, *P < *0.01.

Based on gas chromatography-mass spectrometry (GC-MS) analysis, a total of 158, 158, and 84 metabolites were detected in the aboveground parts, fibrous roots, and root exudates, respectively (Tables S4 to S6). Orthogonal projection to latent structures-discriminant analysis (OPLS-DA) demonstrated that the overall metabolic patterns in the aboveground parts, fibrous roots, and root exudates from foliar-infected plants were distinct from those of uninfected plants (Fig. S3A to C). Most of the metabolites were categorized as alkanes, esters, ketones, sugars and derivatives, amines, alcohols, acids, and amino acids and derivatives. Among them, sugars and derivatives, amines, alcohols, acids, and amino acids and derivatives in the aboveground parts, fibrous roots, and root exudates were all upregulated when plants were infected by *A. panax* (Fig. 4A to C). We further investigated the metabolite changes in root exudates with or without foliar infection by ultraperformace liquid chromatography–quadrupole time of flight tandem MA (UPLC-QTOF-MS/MS) analyses. OPLS-DA and the score plots showed a clear separation in the metabolite accumulation of root exudates under leaves with or without disease infection (Fig. S3D and E). A total of 31 differential metabolites were identified in root exudates (Fig. S3F and G). Most of the metabolites in root exudates categorized as acids and amino acids and derivatives were upregulated, but sugars and derivatives, amines, and nucleosides and derivatives were downregulated when plants were infected by *A. panax* (Fig. 4D).

A total of 38 differential metabolites (variable importance in the projection [VIP], >1; *P* < 0.05) from GC-MS analyses were significantly affected by foliar infection, and most of the metabolites were significantly upregulated in root exudates after foliar infection (Table S7). A total of 16 metabolites (VIP, >1; *P* < 0.05) from UPLC-Q-TOF-MS/MS analysis were significantly affected by foliar infection, and organic acids (palmitic acid, propionic acid, and azelaic acid), amino acids (biocytin and l-serine), and sugars (d-mannose) in root exudates were significantly upregulated after foliar infection. Other metabolites were significantly downregulated, and most of them belonged to nucleosides and their derivatives (Table S7). With combined GC-MS and UPLC-QTOF-MS/MS analyses, a total of 24 metabolites (VIP, >1; *P* < 0.05; fold change, >14 [GC-MS], fold change, >1.4 [UPLC-QTOF-MS/MS]) were significantly upregulated by foliar infection, from which we selected the differential metabolites belonging to organic acids, amino acids, and sugars for subsequent analysis (Table S7; Fig. 4E).

### The metabolites upregulated by foliar infection enhanced the resistance of *P. notoginseng* to foliar infection and the survival rate in continuously cultivated soil.

**FIG 5:**
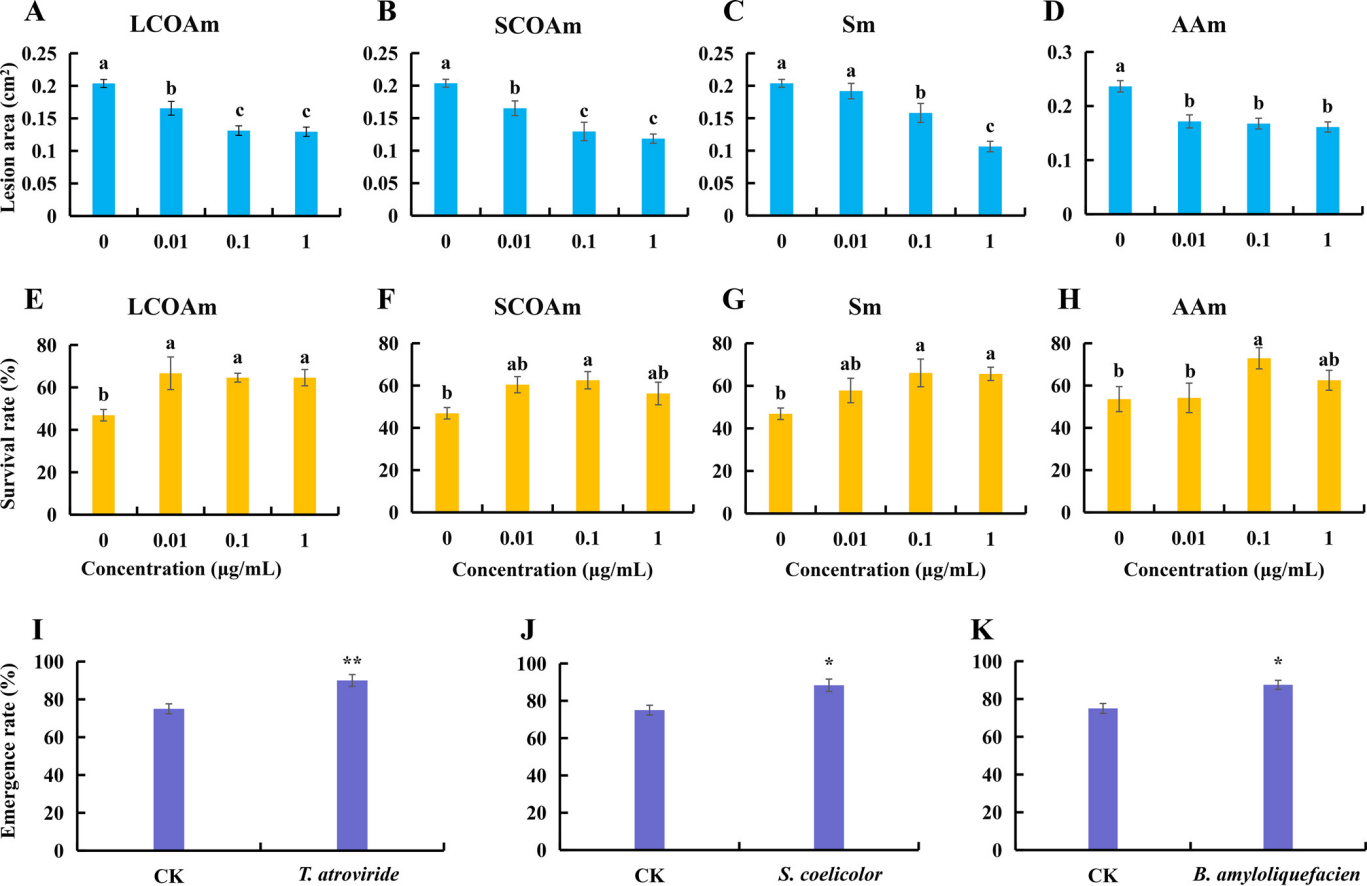
The ability of the upregulated metabolites and microorganisms by foliar infection to alleviate NPSF. (A to D) Effects of the upregulated metabolites added to the rhizosphere soil on the resistance of plants to *A. panax*. (E to H) Effects of the upregulated metabolites added to the continuously cultivated soil on the survival rate of *P. notoginseng*. LCOAm, long-chain organic acid (phthalic acid, palmitic acid, and azelaic acid) mixture; SCOAm, short-chain organic acid (glutaric acid, glyceric acid, dl-beta-hydroxybutyric acid, glycolic acid, and propionic acid) mixture; Sm, sugar (ribose and d-mannose) mixture; AAm, amino acid (l-aspartic acid, alanine, oxoproline and l-serine) mixture. (I to K) Effects of foliar infection-recruited microorganisms added to sterilized soil on the seed germination of *P. notoginseng.* The values represent the means ± SEs. Different letters on the bars indicate significant differences between different treatments (*P < *0.05). *, *P < *0.05; **, *P < *0.01.

The long-chain organic acid mixture (LCOAm), short-chain organic acid mixture (SCOAm), sugar mixture (Sm), and amino acid mixture (AAm) decreased the lesion area on leaves when they were added to the rhizosphere of *P. notoginseng* at concentrations of 0.01, 0.1, and 1 μg/mL (Fig. 5A to D). When we used the metabolite mixture to treat the continuously cultivated soil of *P. notoginseng*, the survival rate was significantly increased by LCOAm, SCOAm, Sm, and AAm (Fig. 5E to H). When foliar infection-enriched microorganisms, including T. atroviride (Fig. 5I), Streptomyces coelicolor (Fig. 5J), and Bacillus amyloliquefaciens (Fig. 5K), were inoculated into the sterilized soil, the emergence rate was significantly increased compared with that of the control.

**FIG 6:**
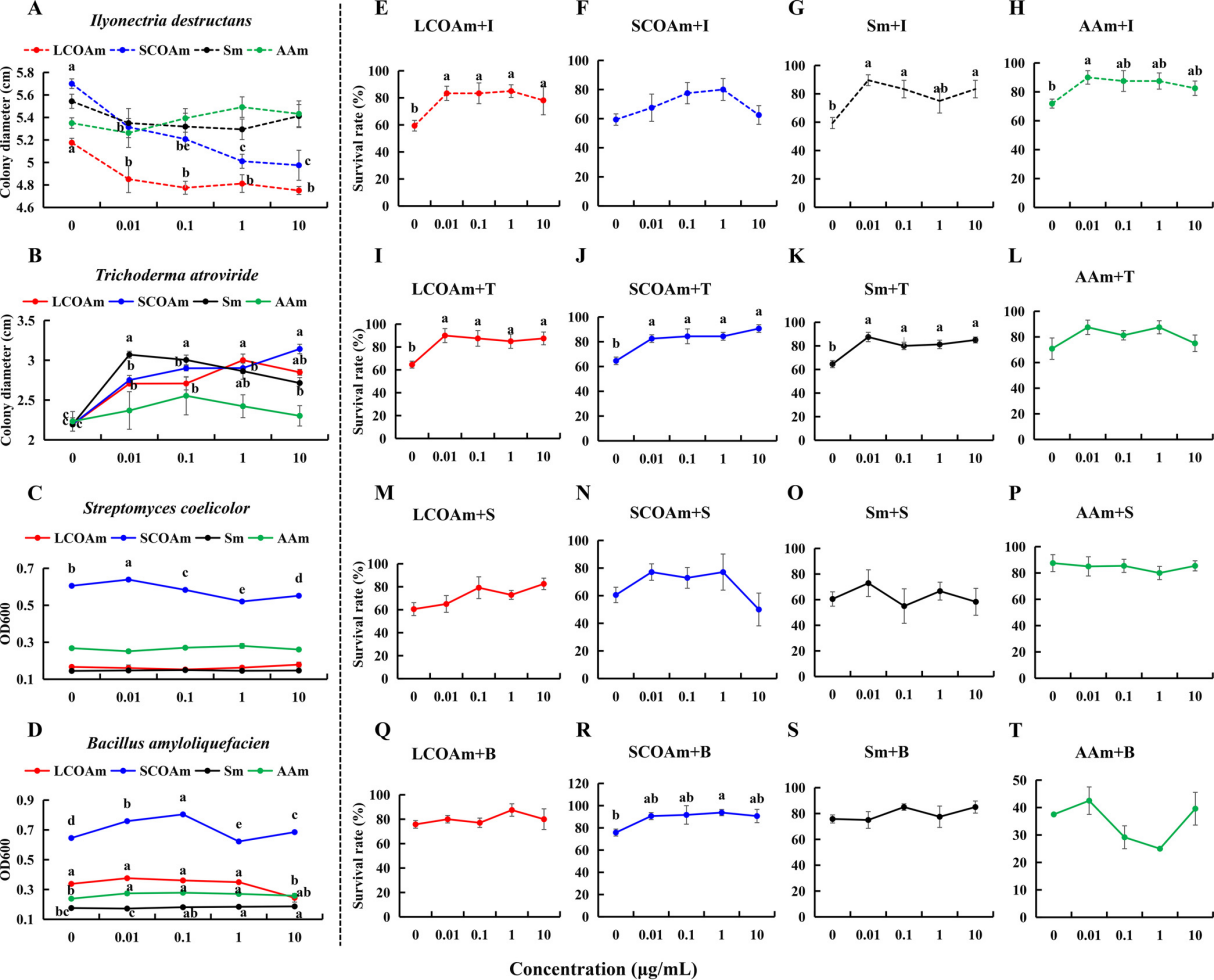
Effects of the root exudate metabolites upregulated by foliar infection on the growth of pathogenic and beneficial microbes as well as their synergistic effect on the survival of *P. notoginseng*. (A to D) Effects of the upregulated root exudate metabolites on the growth of *I. destructans* (A), T. atroviride (B), S. coelicolor (C), and *B. amyloliquefaciens* (D). (E to T) Effects of *I. destructans*, T. atroviride, S. coelicolor, or *B. amyloliquefaciens* in collaboration with a long-chain organic acid (phthalic acid, palmitic acid, and azelaic acid) mixture (LCOAm), a short-chain organic acid (glutaric acid, glyceric acid, dl-beta-hydroxybutyric acid, glycolic acid, and propionic acid) mixture (SCOAm), a sugar (ribose and d-mannose) mixture (Sm), and an amino acid (l-aspartic acid, alanine, oxoproline, and l-serine) mixture (AAm) in root exudates on the survival rate of *P. notoginseng*. I, *I. destructans*; T, T. atroviride; S, S. coelicolor; B, *B. amyloliquefaciens*. The values represent the means ± SEs. Different letters on the line chart indicate significant differences between different concentrations (*P < *0.05).

The amendment of medium with the upregulated metabolite mixture inhibited the growth of pathogenic *I. destructans* (Fig. 6A). The long organic acid mixture (LCOAm) and short organic acid mixture (SCOAm) significantly inhibited the growth of *I. destructans* (Fig. 6A). In particular, the inhibition of SCOAm was dose dependent (Fig. 6A). However, the upregulated metabolite mixture stimulated the growth of beneficial microorganisms. LCOAm, SCOAm, Sm, and AAm significantly promoted the growth of T. atroviride (Fig. 6B). SCOAm at a low concentration (0.01 μg/mL) significantly promoted S. coelicolor growth (Fig. 6C). SCOAm, Sm, and AAm significantly promoted the growth of *B. amyloliquefaciens* (Fig. 6D). A pot experiment further confirmed that LCOAm, Sm, or AAm promoted the survival of *P. notoginseng* when they were added to sterilized natural soil inoculated with pathogenic *I. destructans*, although the difference for the short-chain organic acid mixture was not significant (Fig. 6E to H). LCOAm, SCOAm, and Sm cooperated with T. atroviride and promoted the survival of *P. notoginseng* in sterilized natural soil (Fig. 6I to L). Synergism of these differential metabolites with S. coelicolor was not obvious (Fig. 6M to P). SCOAm at a concentration of 1.0 μg/mL synergized with *B. amyloliquefaciens* (Fig. 6R) and significantly promoted the survival rate of *P. notoginseng*.

## DISCUSSION

Plant growth can lead to the accumulation of soilborne pathogens in rhizosphere soil, thus building negative feedback loops between the soil and plants. When challenged by pathogen infection, plants can recruit beneficial microbes to help themselves (20–27). In this study, we found that belowground plant parts modified the rhizosphere soil microbial community and function by changing plant metabolism and secretion when aboveground leaves were infected by pathogens, eventually changing the plant-soil feedback from negative to positive. This phenomenon reveals an exciting strategy for us to achieve belowground soilborne disease management by manipulating the aboveground state.

### Foliar infection drives changes in plant and soil feedback by modifying the microbiome.

*P. notoginseng* experiences a strong NPSF due to the buildup of soilborne pathogens but suppression of corresponding antagonistic microbes (32, 33). Foliar infection of *P. notoginseng* by *A. panax* could drive the plant-soil feedback from negative to positive (Fig. 1B). Interestingly, the shift was inconspicuous after soil sterilization at 121°C for 20 min (Fig. 1B), which suggested that the change in PSF was correlated with the shift in rhizosphere soil microbial communities. At the phylum level, *P. notoginseng* significantly enriched the relative abundance of *Ascomycota* and *Proteobacteria* but suppressed *Basidiomycota*, *Zygomycota*, *Planctomycetes*, and *Cyanobacteria* compared with no-plant soil (Fig. S2A and C). These data were consistent with previous studies, as the taxa changed in rhizosphere soil at the phylum level with the growth of *P. notoginseng* and other ginseng plants in the *Panax* genus (33–35). However, it is interesting that aboveground infection by foliar pathogens induced some rhizosphere fungi and bacteria at the genus level to show a completely opposite trend compared with that seen for uninfected *P. notoginseng* (Fig. 2 and 3). The relative abundance of the soilborne pathogen *I. destructans* was significantly suppressed, but the beneficial fungi *T. harzianum* and T. atroviride, as well as the bacteria *Bacillus* and *Streptomyces* were significantly enriched in rhizosphere soil after plants were infected by foliar pathogens (Fig. 2C and 3C). *I. destructans* was identified as a soilborne pathogen in this and previous studies, and it causes severe root rot in ginseng plants (30, 36). Previous studies have shown that *Trichoderma*, *Bacillus*, and *Streptomyces* are beneficial taxa with biocontrol abilities (33, 37–40). In this study, we also confirmed that the enrichment of *Trichoderma* spp., *Bacillus* spp., and *Streptomyces* spp. from rhizosphere soil by foliar infection not only resulted in antagonistic activity against *I. destructans* but also enhanced the resistance of *P. notoginseng* against foliar pathogen infection (Fig. 2F and G; Fig. 3D and E). A growing body of research supports that plants under biotic or abiotic stress enrich and sustain specific beneficial microbes from rhizosphere soil to help them deal with the stress (24, 25, 41). Disease-suppressive soil formation has been demonstrated as the recruitment by plants of antagonistic microflora under the stress of long-term soilborne pathogen infection (23). Moreover, beneficial microbe recruitment under foliar biotic stress (pathogen infection, wounds made by pests, etc.) could help plants resist diseases by activating induced systemic resistance (ISR) (25, 27, 42). Therefore, soilborne pathogen suppression and beneficial microbe recruitment by *P. notoginseng* after foliar infection could drive plant and soil feedback from NPSF to PPSF through antagonism against soilborne pathogens and ISR.

### Foliar infection modifies root exudation to suppress pathogens but recruit beneficial microbes.

The rhizosphere microbial communities are mainly shaped by root exudates (43), which are affected by biotic and abiotic factors (44, 45). Many studies have found that some components in root exudates of plants can promote the growth of soilborne pathogens (46–48). Our previous study found that benzoic acid, phthalic acid, and lauric acid in root exudates of *P. notoginseng* had the effects of promoting and inhibiting soilborne pathogens at low and high concentrations, respectively, while cellobiose and maltotriose promoted spore germination of *I. destructans* and had chemotactic effects on germ tube elongation of Fusarium solani (49). The ginsenosides at low concentrations (0.2 to ~20.0 mg/L) secreted from the roots of *P. notoginseng* had a significant growth-promoting effect on Phytophthora cactorum, Pythium irregulare, F. solani, Fusarium oxysporum, and *I. destructans* but inhibited the growth of the beneficial fungus Trichoderma hamatum (50, 51). These interactions between plants and microbes mediated by root exudates resulted in negative plant-soil feedback. However, pathogen infection- or insect feeding-induced changes in root exudates were reported to recruit beneficial microbes to help the plants directly or indirectly (24, 26, 27, 52). In this study, we found that foliar infection enhanced the secretion of short- and long-chain organic acids, sugars, and amino acids (Fig. 4). Interestingly, exogenous addition of a long-chain organic acid mixture, short-chain organic acid mixture, sugar mixture, or amino acid mixture into soils induced plant resistance and alleviated NPSF (Fig. 5). Further experiments revealed that these metabolites in root exudates enhanced by foliar infection could elicit disease suppression by promoting the growth of the beneficial microbes T. atroviride, *B. amyloliquefaciens*, and S. coelicolor but suppressed the growth of the root rot pathogen *I. destructans* (Fig. 6). The pot experiment further confirmed that these metabolites in root exudates enhanced by foliar infection synergized with T. atroviride, *B. amyloliquefaciens*, and S. coelicolor but suppressed *I. destructans* to promote the survival rate of *P. notoginseng* (Fig. 6). The dual function of root exudates in relation to microbes could be explained by the fact that different soil microorganisms have different sensitivities to root exudates. Many studies have shown that some substances, such as defensin peptides secreted by roots, could provide a chemical defense against pathogen infection (53). Phenolic acids or palmitic acid in root exudates could inhibit the growth of the soilborne pathogens Fusarium oxysporum and Verticillium dahliae (54, 55). Azelaic acid can induce plant resistance to pathogens (56). dl-beta-hydroxybutyric acid has a definite inhibitory effect on inflammation (57). Therefore, root exudates upregulated by foliar infection play an important role in defense against pathogen infection.

Moreover, foliar infection-induced root exudates selectively inhibited the soilborne fungal pathogen *I. destructans*, while the antagonistic and ISR-inducing rhizobacteria T. atroviride, *B. amyloliquefaciens*, and S. coelicolor were highly tolerant of these antimicrobial effects (Fig. 6A to D). Root exudates can serve as carbon and nitrogen substrates for microbial growth (58, 59) or act as signal molecules for microbial aggregation in the rhizosphere (60). The promotion of T. atroviride, *B. amyloliquefaciens*, and S. coelicolor by long-chain organic acids, short-chain organic acids, sugar mixtures, and amino acids supports their utilization in root exudates (Fig. 6B to D). Therefore, foliar infection-upregulated root exudates play a dual function in the suppression of pathogens but recruitment of beneficial microbes in the rhizosphere. These phenomena are widely found in the rhizosphere. Some phytoalexins in root exudates, such as coumarins (61), scopoletin (62), malic acid (59), benzoxazinoids (63), and camalexin (64), also showed dual roles in the suppression of pathogens but recruitment of beneficial microbes in the rhizosphere. The enrichment and suppression of specific rhizosphere microbiomes after foliar infection could be partly explained by differences in the utilization and detoxification of root exudates (48), but the underlying mechanism deserves further exploration.

To perceive microbial signals in an effective and timely manner, plants have evolved a multilayered detection system that leads, depending on the trigger, to the activation of downstream defense responses (65). Based on their timing, the activated immune responses range from instant (medium alkalization, oxidative burst [reactive oxygen species (ROS)], protein phosphorylation) and early (ethylene biosynthesis, defense gene activation) to late (callose deposition and growth inhibition) (66). The whole process is divided into pathogen-associated molecular pattern (PAMP)-triggered immunity (PTI) and effector-triggered immunity (ETI) (67). Plants use these innate types of immunity of their own cells to make the whole plant systematically acquire resistance (SAR) or induce systemic resistance (ISR) (68). Many studies have shown that roots can perceive microbe-associated molecular patters (MAMPs) and generate MAMP-specific responses such as callose deposition, camalexin biosynthesis, and induction of defense-related genes similar to leaves (69–72). In these processes, signaling pathways of the defense hormones salicylic acid (SA) and jasmonic acid (JA) play an important role (42, 73, 74). Some researchers showed SA-dependent signaling is generally effective against biotrophic pathogens, whereas JA/ethylene (ET)-dependent signaling is generally effective against necrotrophic pathogens in interactions between *Arabidopsis* and its pathogens (73, 75). It was found that SA-, Methyl Jasmonate- (MeJA-) and Nitric Oxide- (NO-) elicited roots increased the root exudation of phytochemicals, including the amino acid, sugar, and inorganic solute transporters (76). In addition, plants will regulate the expression of corresponding stress-resistant genes and proteins in cells through the signal transduction, such as Ca^2+^, finally changing metabolism and root secretion to respond to environmental shifts after sensing the stress signal (77–80). These indicate that plants can change metabolism and secretion by regulating the pathways related to the synthesis of hormones and other signal to help themselves resist the damage of adversity. In this study, we described that foliar infection could change the rhizosphere microbial community by driving root exudate shifts, but its underlying mechanism requires further study.

**FIG 7:**
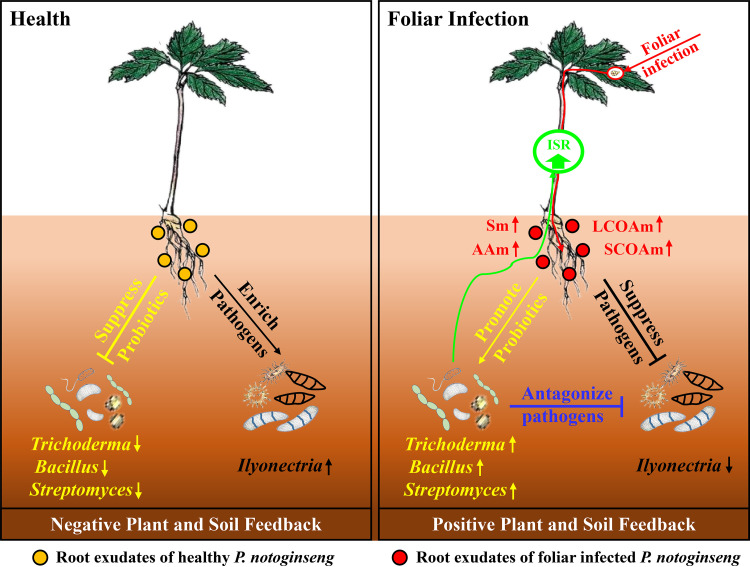
Mechanistic model of foliar infection by pathogens changing the plant-soil feedback from negative to positive through the root exudate modified rhizosphere microbiome. LCOAm, long-chain organic acid (phthalic acid, palmitic acid, and azelaic acid) mixture; SCOAm, short-chain organic acid (glutaric acid, glyceric acid, dl-beta-hydroxybutyric acid, glycolic acid, and propionic acid) mixture; Sm, mixture of sugars (ribose and d-mannose); AAm, amino acid (l-aspartic acid, alanine, oxoproline, and l-serine) mixture. Up arrows represent upregulation. Down arrows represent downregulation.

**Conclusion.** The aboveground parts of plants infected by foliar pathogens changed plant metabolism and root secretion. In particular, the upregulated organic acids, sugars, and amino acids in root exudates mediated changes in the microbiome in the rhizosphere, such as suppression of soilborne pathogens and enrichment of beneficial microbes that antagonize soilborne pathogens, and induced plant system resistance, eventually changing the plant and soil feedback from negative to positive (Fig. 7). Therefore, this represents a potential strategy to achieve belowground soilborne disease by manipulating the aboveground state.

## MATERIALS AND METHODS

### Experimental design and sampling.

Natural soils without a *P. notoginseng* cultivation history were sampled from Pinus yunnanensis (3 sites and 3 samples each site), *P. kesiya* (3 sites and 3 samples each site), and *Eucalyptus* (1 site and 3 samples) forests (Table S1). Some of the soils were separately placed in pots, and 10 healthy seeds of *P. notoginseng* were sown in each pot and grown for 6 months. Soil in a pot without seedlings was the no-plant soil treatment. Three treatments, bulk soil without seedlings, seedling inoculation, and noninoculation with *A. panax*, were used in the experiment (Fig. 1A, step 1). In the foliar pathogen infection treatment, an *A. panax* mycelial block (6-mm diameter) was placed face down on a *P. notoginseng* leaf that had a premade wound, and two leaves of each seedling were inoculated. Uninoculated seedlings were wounded by needle only (Fig. 1A, step 1). All pots were prewrapped in clear plastic bags to prevent contamination of the soil by *A. panax.* All pots and plants were placed in transparent boxes and incubated with a photoperiod of 16 h light/8 h dark at 25 ± 2°C while keeping the humidity suitable for pathogen infection (Fig. 1A, step 1). When the leaves showed symptomatic lesions, the rhizosphere soil was sampled following a previously described procedure (33). All root surface soil was removed until the remaining aggregates were within 1 mm from the root surface. Roots were placed in a sterile 50-mL tube containing 40 mL 1× phosphate-buffered saline (PBS) buffer. Tubes were vortexed at maximum speed for 15 s to wash off the rhizosphere soil from the roots. The washing buffer was subjected to centrifugation (12,000 × *g* for 10 min), and the collecting precipitate was defined as the rhizosphere soil. The soil without seedlings was sampled as no-plant soil. Each treatment contained three replicates, and each replication included six pots; all treatments were collected and stored at −80°C for microbial analysis. The intact uninoculated leaves and fibrous roots were collected after washing with pure water and stored at −80°C for metabolic analysis.

### Evaluation of the feedback relationship between *P. notoginseng* and foliar pathogen infection-conditioned soil.

The remaining soil after removal of the plants and the no-plant soil in the pots were used to evaluate the feedback relationship between *P. notoginseng* and the foliar infection-conditioned soil according to previous methods (81) (Fig. 1A, step 2). Briefly, all the remaining soil was divided equally in two parts. One part was sterilized at 121°C for 20 min, and the other was not treated. The soils were placed into the seedling nursing trays (50 cells per tray, 5.0 cm by 5.0 cm by 8.0 cm per cell). Sterilized seeds with 1% sodium hypochlorite were planted into the cells of seedling nursing trays with a photoperiod of 16 h light/8 h dark at 25 ± 2°C. The seed emergence rate in soil was recorded, and feedback ratios were calculated for each replicate pair as (Ai-Bi)/maximum (Ai, Bi), where A and B are emergence rates obtained in soil treatments with or without sterilization. Some pots with the unsterilized remaining soils in all treatments were used for evaluating the disease resistance according to previous methods (82). The leaves of seedlings in the foliar pathogen infection-conditioned soil and no-plant soil were inoculated with *A. panax* and incubated with a photoperiod of 16 h light/8 h dark at 25 ± 2°C according to the above-described method (Fig. 1A, step 3). When the leaves showed symptomatic lesions, the diseased spot areas were scanned with an Epson perfection V850 Pro scanner, and their size was estimated using the measuring tool in Adobe Photoshop CS6.

### Microbial community analysis.

Soil genomic DNA was extracted using the Power Soil DNA isolation kit (Mo Bio Laboratories, Inc., Carlsbad, CA, USA) following the manufacturer’s instructions. The fungal genes were amplified with the primers ITS2F (GCATCGATGAACGCAGC) and ITS2R (TCCTCCGCTTATTGATATGC). The bacterial genes were amplified with the primers 341F (CCTAYGGGRBGCASCAG) and 806R (GGACTACNNGGGTATCTAAT). Fungal internal transcribed spacer (ITS) and bacterial 16S rRNA genes in the soil total DNA samples were sequenced using the Illumina HiSeq platform. After the raw reads were spliced using QIIME (83) and quality filtered (84), the retained effective sequences were used for operational taxonomic unit (OTU) clustering and species annotation. OTUs were defined at ≥97% sequence identity using USEARCH. The taxonomic identities were determined according to the Unite (fungi) and Silva (bacteria) databases (85). The data of each sample were then normalized according to the minimum data in the sample, and the annotated proportion of each classification level of OTUs was calculated with MicrobiomeAnalyst (86). All sequences of ITS and 16S rRNA genes can be found in the Sequence Read Archive (SRA) at NCBI (https://trace.ncbi.nlm.nih.gov/Traces/sra) under BioProject number PRJNA838812.

### Identification of the potential functional microorganisms modified by foliar infection.

Bacteria and fungi were isolated on plates according to Luo’s method (33). Briefly, 10 g of the rhizosphere soil sample was added to 90 mL of sterilized water, which was homogenized for 15 min and decimally diluted (from 10^−1^ to 10^−7^). Then, 100 μL of the solutions was plated on nutrient agar (NA) medium (87) and potato dextrose agar (PDA) with 100 μg · mL^−1^ chloramphenicol for the potential functional microorganism’s isolation. After incubation at 25°C for 4 to 5 days, individual colonies on the plates were picked out and inoculated on NA medium (bacteria) or PDA (fungi) to obtain cultures. The foliar infection-modified fungi and bacteria were identified and chosen through the morphological and ITS or 16S rDNA amplification method, respectively (88).

The pathogenicity of four *I. destructans* isolates was determined on *P. notoginseng* roots *in vitro* according to Luo’s method (33). A total of 48 roots were inoculated for each isolate, with a noncolonized agar block as a control. After 5 days of inoculation, pathogens were isolated from every root with symptomatic lesions.

Induced systemic resistance (ISR) of plants by foliar infection-modified *Trichoderma* spp., *Streptomyces* spp., and *Bacillus* spp. was tested. Ten healthy seedlings were planted in each pot with sterilized soil. After 1 month, a pot was inoculated with 30 mL (10^6^ CFU · mL^−1^) of the isolate suspension for 24 h. The treated pots were used to evaluate black spot disease resistance according to the above-described method (Fig. 1A, step 3). Each treatment contained 12 pots.

Antagonistic activity of the above-mentioned beneficial microbes against *I. destructans* was tested in a dual culture following the method described in a previous study (89). Briefly, the mycelial block was cocultured with beneficial microbes on the same PDA plate. Plates with only pathogens grown on PDA were used as controls. Four replicate plates were used per treatment. All treatments were incubated at 25°C for 5 days. The mycelial growth of the pathogen was determined by measuring the colony semidiameter. The growth inhibition rate was calculated as follows:

Growth inhibition rate (%)=100×(Radial growth of control−Radial growth of treated sample)/Radial growth of control

The effects of foliar infection-modified microorganisms on the emergence rates of *P. notoginseng* in sterilized soil were assessed in a pot experiment. Ten surface-sterilized seeds were sown in each pot with the sterilized soil and inoculated with 50 mL (10^6^ CFU · mL^−1^) of the above isolate suspension. Pots without inoculation were used as the blank control. The pots were placed in a growth chamber with a photoperiod of 16 h light/8 h dark at 25 ± 2°C. Each treatment contained three replicates, and each replication included six pots. Then, 3 months after treatment, the emergence rates were recorded.

Emergence rate (%)=100×Emerged seedlings/Total seeds in each treatment

### GC-MS analyses of the above- and belowground tissues after foliar infection.

To explain how foliar infection of *P. notoginseng* changes plant metabolism, the aboveground parts and fibrous roots of uninoculated and inoculated plants were analyzed by gas chromatography-mass spectrometry (GC-MS). Derivatization of the sample was performed according to a previous method (90), and 80 μL of the supernatant transferred to vials was detected by GC-MS (91). Briefly, detection was performed using a gas chromatograph-mass spectrometer (GCMS-QP2010 Ultra, Shimadzu, Japan) with an SH-Rxi-5Sil MS column (30.0 m by 0.25 mm by 0.25 μm). An Abf Converter, MS-DIAL (92), Shimadzu offline software, and the NIST 14 library were used for peak identification and related data generation. Data were normalized on MetaboAnalyst 4.0 (93). SIMCA-P 14.1 (Umetrics, Umea, Sweden) was used for orthogonal projection to latent structures-discriminant analysis (OPLS-DA). The differential metabolites were screened based on their variable importance in the projection (VIP) and *P* value (VIP >1; *P* < 0.05).

### Root exudate collection and analyses by GC-MS and UPLC-QTOF/MS.

Root exudates were collected according to previous methods with some modifications (94). Briefly, eight clean healthy seedlings were transferred into 60 mL sterile distilled water in a glass pot wrapped in tinfoil. The free space at the pot mouth was covered with sealing film to prevent contamination. The leaves of seedlings were inoculated following the method described above. Each treatment contained 8 biological replicates, and one replicate was 6 pots. When the leaves showed symptomatic lesions, the solution in the pots was filtrated with filter paper and 0.22 μm hydrophilic membranes and then concentrated to dry matter under reduced pressure. The dry matter was defined as root exudates and stored at −80°C and then analyzed by GC-MS and UPLC-QTOF/MS.

Root exudates were simultaneously analyzed by GC-MS according to the above-described method and UPLC-QTOF/MS as described in a previous study (95). Briefly, metabolic profiling of root exudate samples was performed on an Agilent 1290 Infinity LC system (Agilent Technologies, Santa Clara, CA, USA) with an Acquity UPLC ethylene-bridged hybrid (BEH) amide chromatographic column (Waters, 1.7 μm, 2.1 mm by 100 mm) coupled with an triple TOF 5600 system (AB Sciex, Framingham, MA, United States). The original data were converted into mzxml format by ProteoWizard, and then the XCMS program was used for peak alignment, retention time correction, and peak area extraction. Accurate mass number matching (<25 ppm) and secondary spectral matching were used for metabolite structural identification when searching the self-built database from Shanghai Applied Protein Technology Co., Ltd. For the data extracted by XCMS, the ion peak of the group summation >2/3 was deleted. The data analysis was the same as that described above.

### Functional evaluation of root exudate metabolites upregulated by foliar infection.

Ten healthy seeds were sown in each pot with natural soil. After 6 months of growth under natural conditions, a 50-ml long-chain organic acid (phthalic acid, palmitic acid, azelaic acid) mixture (LCOAm), short-chain organic acid (glutaric acid, glyceric acid, dl-beta-hydroxybutyric acid, glycolic acid, and propionic acid) mixture (SCOAm), sugar (ribose and d-mannose) mixture (Sm), or amino acid (l-aspartic acid, alanine, oxoproline, and l-serine) mixture (AAm) with final concentrations of 0.01, 0.1, or 1 μg/mL was added to each pot and maintained for 24 h. Methanol (0.1%) was used as a control. The corresponding characteristics of the metabolites can be found in Table S2. The treated pots were used to evaluate the effects of upregulated metabolites on the resistance of *P. notoginseng* against *A. panax* according to the above-described method (Fig. 1A, step 3). Each treatment contained 12 pots.

Effects of the upregulated metabolites on the survival rate of *P. notoginseng* were tested in pots with consecutively cultivated soil. First, 50 mL of LCOAm, SCOAm, Sm, or AAm at final concentrations of 0.01, 0.1 or 1 μg/mL was added to each pot for 3 days. Methanol (0.1%) was used as a control. Then, eight healthy *P. notoginseng* seedlings were sown in each pot. All pots were placed in a growth chamber with a photoperiod of 16 h light/8 h dark at 25 ± 2°C. Each treatment contained eight pots. Then, 3 months after treatment, the seedling survival rate was recorded.

Seedling survival rate (%)=100×Living seedlings/Total seedlings in each treatment

The effect of upregulated metabolites on the growth of mycelia of *Trichoderma atroviride* or *I. destructans* was determined by the colony diameter method in PDA medium amended with LCOAm, SCOAm, Sm, or AAm at final concentrations of 0.01, 0.1, 1.0, and 10 μg/mL (96). The effects of upregulated metabolites on the growth of Bacillus amyloliquefaciens and Streptomyces coelicolor were measured following a published procedure (96). Briefly, the above-mentioned metabolites were added into NA liquid medium with 1 × 10^6^ CFU/mL to reach the final concentrations of 0.01, 0.1, 1.0, and 10 μg/mL. The suspensions were placed in 96-well microplates and measured at 600 nm to determine proliferation (optical density at 600 nm [OD_600_] reads) with a VersaMax microplate reader (Molecular Devices, Sunnyvale, CA, USA).

A pot experiment was performed to test the synergistic effects of *I. destructans*, T. atroviride, *B. amyloliquefaciens*, and S. coelicolor with the upregulated metabolites in root exudates. Eight healthy *P. notoginseng* seedlings were planted in each pot with sterilized soil. Solutions of LCOAm, SCOAm, Sm, or AAm were formulated to the desired target concentrations of 0.01, 0.1, 1.0, or 10 μg/mL, respectively. Then, 50 mL of different metabolite mixtures in combination with T. atroviride (10^6^ CFU/mL), *I. destructans* (10^6^ CFU/mL), *B. amyloliquefaciens* (OD_600_, 0.5), or S. coelicolor (OD_600_, 0.5) was added to the pots. Pots with the same volume of water with/without 0.1% methanol were taken as the blank controls. The pots were placed in a growth chamber with a photoperiod of 16 h light/8 h dark at 25 ± 2°C. Six replicates were prepared. Then, 3 months after inoculation, the survival rate was recorded.

### Statistical analysis.

Data were analyzed using SPSS 17.0 software (SPSS, Inc., USA) and Prism 7.0 software (GraphPad, Inc., USA). One-way analysis of variance (ANOVA) and Duncan’s multiple-range test (*P < *0.05) were used for statistical analysis. Student’s *t* test was used to analyze the mean separation among treatments.

### Data availability.

All raw sequencing data have been submitted to the NCBI Sequence Read Archive (SRA) database under BioProject number PRJNA838812.
