# Supplementary material for: Foliar Pathogen Infection Manipulates Soil Health through Root Exudate-Modified Rhizosphere Microbiome
Source: Microbiol Spectr. 2022 Nov 29;10(6):e02418-22. doi: 10.1128/spectrum.02418-22 (PMC9769671; doi:10.1128/spectrum.02418-22)
Supplement: Supplemental file 1 — Supplemental material. Download spectrum.02418-22-s0001.pdf, PDF file, 0.9 MB [file spectrum.02418-22-s0001.pdf]

Supplementary material

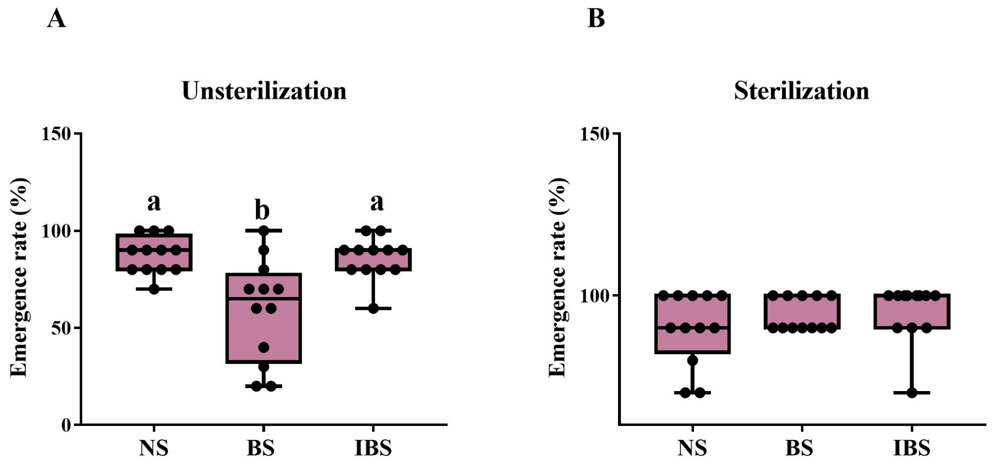

Figure S1 Emergence rate of *P. notoginseng* seedlings growing on pathogen-conditioned and corresponding control soils without (A) or with (B) sterilization. NS represents emergence rate in no-planted soil. BS represents emergence rate in bulk soil. IBS represents emergence rate in pathogen-conditioned soil. Different letters on the bars indicate significant differences between different treatments ($p < 0.05$).

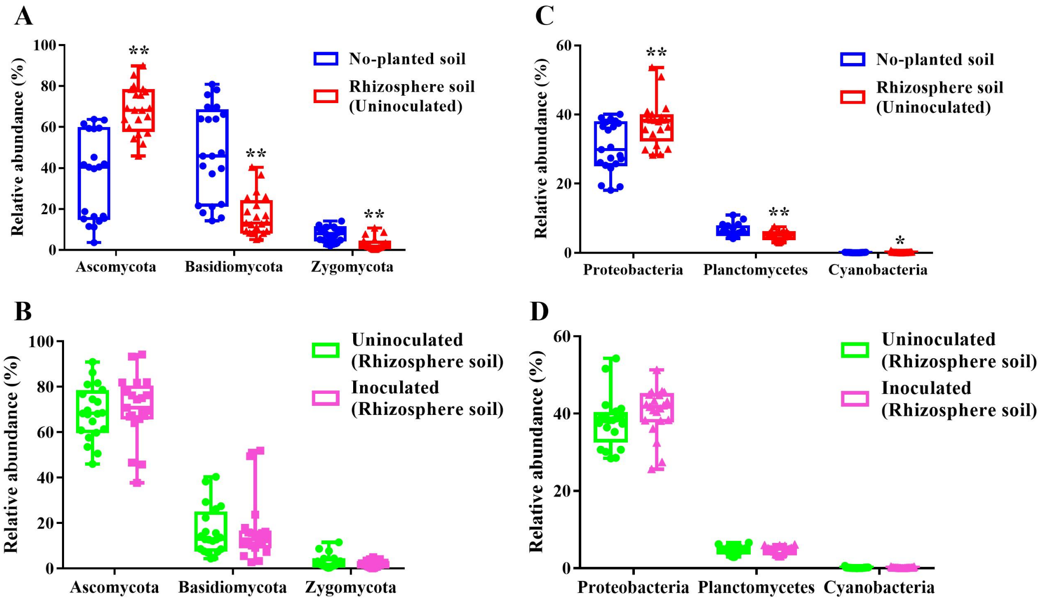

Figure S2 Comparison of fungal (A, B) and bacterial (C, D) abundance differences in no-planted, uninoculated and inoculated *P. notoginseng* rhizosphere soils at the phylum level.

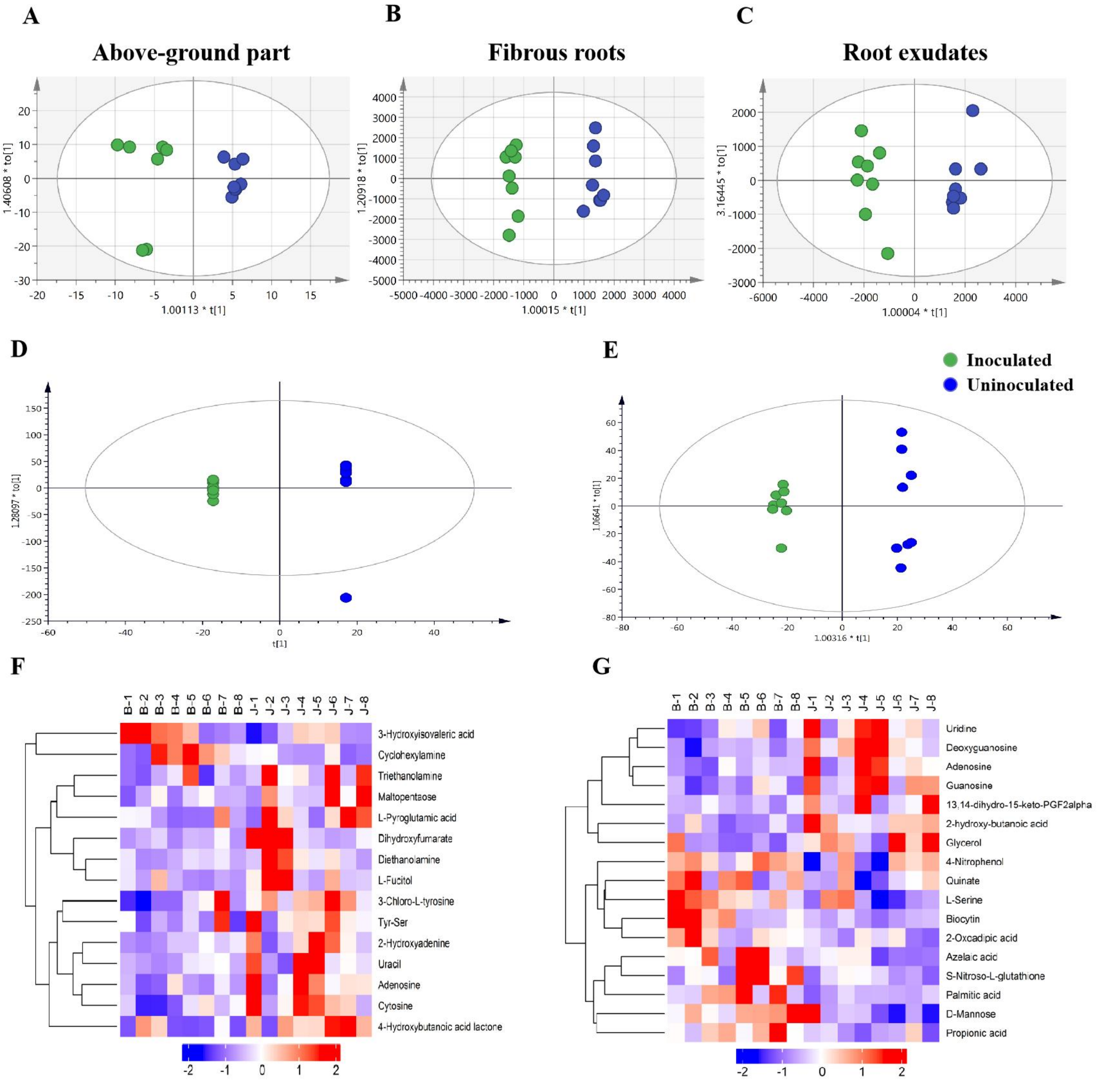

Figure S3 OPLS-DA score plots of aboveground parts (A), fibrous roots (B) and root exudates (C) when *P. notoginseng* leaves are uninoculated and inoculated based on GC–MS analysis. Positive (D) and negative (E) ion mode OPLS-DA score chart for root exudates based on UPLC-Q-TOF-MS/MS analysis. Hierarchical clustering of differential metabolites with positive (F) and negative (G) ion patterns in root exudates based on UPLC-Q-TOF-MS/MS analysis.

Table S1 Characteristics of the soil samples

Tree type	Sample site	Altitude (m)	Alkali-hydrolyzable nitrogen (mg/kg)	Available phosphate (mg/kg)	Available potassium (mg/kg)	Electrical conductivity (μS/cm)	Organic matter (g/kg)	pH
Pinus yannanensis	Niner（NE）	1630	175.7±8.9	3.7±1.2	122.0±4.0	87.37±24.0	34.6±6.1	5.96±0.1
	Lancang（LC15）	1510	334.1±17.4	8.0±2.6	114.1±1.2	50.23±2.3	72.8±9.3	5.83±0.2
	Lancang（LC18）	1890	320.4±8.8	7.7±1.7	114.0±1.9	72.33±17.8	68.2±8.4	5.11±0.1
Pinus kesiya	Wenshan（S）	1560	224.5±11.8	3.7±2.3	397.9±25.3	68.50±1.9	49.7±7.4	5.82±0.1
	Wenshan（U）	1310	226.1±7.4	3.7±1.7	244.9±3.3	82.20±0.8	60.4±8.6	6.70±0.1
	Lijiang (H)	2080	350.7±52.7	6.1±0.7	404.7±31.5	75.45±1.3	45.6±3.2	6.61±0.1
Eucalyptus	Lancang (A)	1890	244.5±9.6	7.3±1.8	153.1±3.2	73.57±17.6	45.6±4.6	6.14±0.1

Table S2 The remarkable metabolites in *P. notoginseng* root exudates

Compound	Molecular formula	CAS	Purity	Company
Phthalic acid	$C_8H_6O_4$	88-99-3	99.50%	Shanghai Yien Chemical Technology Co., Ltd.
Palmitic acid	$C_{16}H_{32}O_2$	57-10-3	99.95%	Beijing Solarbio science &Technology Co., Ltd.
Azelaic acid	$C_9H_{16}O_4$	123-99-9	99%	Shanghai Macklin Biochemical Co., Ltd.
Glutaric acid	$C_5H_8O_4$	110-94-1	99%	Shanghai Yien Chemical Technology Co., Ltd.
Glyceric acid	$C_3H_6O_4$	473-81-4	95%	Shanghai Yien Chemical Technology Co., Ltd.
DL-beta-Hydroxybutyric acid	$C_4H_8O_3$	300-85-6	98%	Alfa Aesar (China) Chemical Co., Ltd.
Glycolic acid	$C_2H_4O_3$	79-14-1	98%	Shanghai Yien Chemical Technology Co., Ltd.
Propionic acid	$C_3H_6O_2$	79-09-4	99.50%	Shanghai Yien Chemical Technology Co., Ltd.
D-Mannose	$C_6H_{12}O_6$	3458-28-4	99%	Shanghai Macklin Biochemical Co., Ltd.
Ribose	$C_5H_{10}O_5$	50-69-1	98%	Shanghai Yien Chemical Technology Co., Ltd.
L-Serine	$C_3H_7NO_3$	56-45-1	99%	Shanghai Yien Chemical Technology Co., Ltd.
L-Aspartic acid	$C_4H_7NO_4$	56-84-8	99%	Shanghai Yien Chemical Technology Co., Ltd.
Alanine	$C_3H_7NO_2$	338-69-2	99%	Shanghai Yien Chemical Technology Co., Ltd.
Oxoproline	$C_5H_7NO_3$	98-79-3	99%	Shanghai Yien Chemical Technology Co., Ltd.

Table S3 Fungal and bacterial communities sequencing statistics

Class	SampleID	Raw Tags	Clean Tags	Effective Tags
	NE31	186368	185508	182278
	NE32	220416	220001	218565
	NE33	191858	190970	190378
	NE3n1	90390	90225	89765
	NE3n2	93802	93578	93326
	NE3n3	78204	77935	77639
	NE3i1	206980	206507	205099
	NE3i2	190195	189578	188817
	NE3i3	98048	97798	96901
	LC151	80443	79071	79026
	LC152	78289	77865	77834
	LC153	81690	81174	81023
	LC15n1	98016	97576	97381
	LC15n2	81655	81004	80898
	LC15n3	90295	89752	89302
	LC15i1	114053	113751	113721
	LC15i2	101761	100936	100674
	LC15i3	96750	96496	96083
	LC181	100216	99739	99178
	LC182	99352	98787	98605
Fungi	LC183	106869	88245	88205
	LC18n1	101560	101213	100602
	LC18n2	113791	113397	112475
	LC18n3	101188	100685	99651
	LC18i1	93827	93378	92716
	LC18i2	89285	88890	88854
	LC18i3	91076	90697	90410
	S1	102057	101554	100850
	S2	102267	102009	101474
	S3	90949	90337	89577
	Sn1	95224	94989	93509
	Sn2	94242	93800	93044
	Sn3	106212	105971	105746
	Si1	97560	97345	96322
	Si2	107559	107355	105486
	Si3	95602	95359	94996
	U1	75988	75641	75597
	U2	94660	94190	94063
	U3	101828	101355	101008
	Un1	108859	108489	108120
	Un2	116317	115727	115585

	Un3	102467	102237	101965
	Ui1	88161	87860	87775
	Ui2	103541	103258	101240
	Ui3	133501	132920	131941
	H1	66308	66116	65921
	H2	82492	82256	82144
	H3	82634	82415	82174
	Hn1	92257	91734	89742
	Hn2	97350	97104	95936
	Hn3	86462	86189	85595
	Hi1	84486	84155	83516
	Hi2	94445	94328	92402
	Hi3	92957	92687	91939
	A1	150983	149864	149584
	A2	166916	165965	165725
	A3	67650	67381	67291
	An1	79765	79490	79472
	An2	99920	99533	99403
	An3	102580	102150	102008
	Ai1	113400	113297	111834
	Ai2	100128	75363	75177
	Ai3	113101	112801	112646
Bacteria	NE31	140177	138968	123235
	NE32	138817	137795	126053
	NE33	158533	157044	140207
	NE3n1	75975	75446	60832
	NE3n2	79833	79189	69589
	NE3n3	69491	68953	60835
	NE3i1	150475	149141	131614
	NE3i2	141005	139849	127306
	NE3i3	70752	70219	59891
	LC151	79697	79098	77829
	LC152	72057	71492	71177
	LC153	75044	74468	73140
	LC15n1	85154	84567	75626
	LC15n2	70921	70328	68532
	LC15n3	76427	75884	70928
	LC15i1	68925	68372	67751
	LC15i2	83894	83792	79684
	LC15i3	85080	84484	81012
	LC181	62526	62151	61617
	LC182	65511	65082	61510
	LC183	66374	65996	64330
	LC18n1	87980	87422	77716

LC18n2	95134	94468	82316
LC18n3	80633	80136	70662
LC18i1	60194	59797	51948
LC18i2	71457	71014	64683
LC18i3	83431	82840	73097
S1	71483	71000	66366
S2	84134	83590	77092
S3	80253	79665	73537
Sn1	75898	75285	70541
Sn2	86316	85669	77359
Sn3	74824	74296	67477
Si1	85097	84518	75415
Si2	85106	84588	70502
Si3	69903	69443	62828
U1	57523	56955	51749
U2	64944	64294	57842
U3	63868	63221	56371
Un1	76885	76018	67921
Un2	74661	73893	67167
Un3	68653	67978	63309
Ui1	53389	52879	47905
Ui2	87275	86464	76090
Ui3	80760	80127	73251
H1	77870	77109	72214
H2	75343	74639	68914
H3	90049	89127	81481
Hn1	73163	72449	66269
Hn2	78459	77709	71152
Hn3	76971	76208	68697
Hi1	80238	79510	72228
Hi2	89846	88943	79557
Hi3	89073	88192	78602
A1	71303	70836	68975
A2	63397	62960	59851
A3	55242	54866	54499
An1	77564	77016	70161
An2	102580	101588	94560
An3	65772	65209	59194
Ai1	59572	59206	51947
Ai2	62206	61817	61013
Ai3	69970	69504	59308

Note: NE3, LC15, LC18, S, U, H and A represents the soils in sample sites from *Pinus yannanensis, Pinus kesiya and Eucalyptus* forests, respectively. NE3n, LC15n, LC18n, Sn, Un, Hn and An represents the rhizosphere soils after planting *Panax*

notoginseng respectively. NE3i, LC15i, LC18i, Si, Ui, Hi and Ai represents the rhizosphere soils after planting *Panax notoginseng* with foliar infection respectively.

Table S4 A total of 158 metabolites detected in aboveground parts of *P. notoginseng* based on GC-MS analysis

Metabolite name	Peak height (Inoculated)	Peak height (Uninoculated)	Metabolite name	Peak height (Inoculated)	Peak height (Uninoculated)
Quinic acid	89,896.93	25,441.98	Citric acid	2,985,845.98	2,416,976.50
D- (+)-Galacturonic acid	13,498.57	6,373.46	Beta-sitosterol	13,180.21	2,950.17
Glycolic acid	47,976.54	20,202.53	Glycyl proline	50,696.24	53,096.32
Phytosphingosine	130,826.37	50,137.16	Valine	269,088.08	173,229.57
2-Hydroxybutanoic acid	171,392.28	173,688.85	Nonadecane	9,795.15	12,286.90
Isoleucine	250,661.17	109,000.01	D- (+)-Galactosamine	33,899.32	20,939.70
L-Ornithine	19,152.54	7,183.60	Serine	119,070.30	50,395.56
2,4,7-Octanetrione	32,767.34	41,998.14	Phosphate	1,533,577.96	469,963.37
Threonine	75,897.96	37,746.73	Hexose-6-phosphate	226,455.64	51,809.21
1-Heptanol	9,540.44	4,033.44	Heptadecane	14,753.61	20,275.60
Malic acid	2,633,204.68	1,166,570.65	4-Coumaric acid	117,021.07	37,064.14
Tagatose	23,032.62	10,645.43	Glycerol-3-galactoside	16,195.22	5,807.20
Sorbose	1,861,392.41	758,165.05	3-Hydroxy-3-methylglutarate	19,145.58	8,689.04
Psicose	84,081.22	25,157.59	Isocitric acid	4,399,660.03	3,680,927.50
Fructose	1,580,322.05	600,624.33	Oxoproline	1,353,838.11	497,949.09
Arbutin	18,845.89	3,410.09	Malonic acid	192,186.01	78,416.02
4-Aminobutyric acid	776,680.66	328,725.32	5,6-Dihydrouracil	923,590.77	701,952.46
Methanolphosphate	6,430.20	3,162.35	Proline	22,191.90	19,023.73
Lanosterol	23,837.55	5,200.11	3-Phosphoglycerate	36,436.57	7,644.99
Inositol	2,214,692.91	1,298,720.08	Tyrosine	10,666.09	5,063.84
Ascorbic acid	20,799.55	12,612.64	N-Acetyl-d-hexosamine	30,072.23	12,903.81
Aspartate	231,166.84	83,102.54	N-Acetyl galactosamine	929,405.61	378,587.59
2-Aminobutanedioate	53,976.21	25,448.00	N-Acetyl-d-glucosamine	557,616.40	249,917.07
Phenylalanine	145,196.94	84,582.70	Glycerol	286,731.21	176,592.13
Butyl acrylate	127,220.31	172,095.48	Glucono-1,5-lactone	65,620.45	27,988.35
Sucrose	5,108,950.41	4,181,192.75	2-Deoxy-d-glucose	331,055.31	132,556.86
4-Pentenyl isothiocyanate	13,461.93	18,135.95	Rhamnose	154,235.15	58,379.37
Hexadecane	3,174.87	5,451.14	Nicotinic acid	16,843.31	6,720.24
Asparagine	6,338.81	5,423.60	Ribose	20,745.72	7,696.24
Niacinamide	5,893.71	2,260.85	Lyxose	70,728.94	12,827.40
Glycine	8,833.46	11,891.32	3-Amino isobutyric acid	933.96	5,541.88
Decane	16,297.52	20,864.24	Hydroquinone	6,804.89	3,065.60
Melibiose	157,591.75	52,711.52	Stearic acid	192,626.56	141,675.85
Gentiobiose	65,684.39	24,968.91	Alanine	20,609.74	9,615.41

Pinitol	158,265.88	60,202.81	Melezitose	23,488.50	4,920.64
Mucic acid	12,267.78	4,825.14	Ribonic acid	20,650.12	7,461.52
Glucarate	1,941,729.57	930,746.96	Xylonic acid	56,606.97	19,835.27
Sorbitol	41,813.14	10,721.63	Glyceric acid	1,612,550.75	909,044.45
Mannitol	132,809.84	65,230.31	N-Acetylglutamate	21,529.36	4,558.71
Hexitol	87,854.38	259,752.69	Mannonic acid	75,668.30	25,773.97
L-Iditol	69,878.87	21,509.26	Dalactonic acid	72,492.58	30,789.66
Nonanoric acid	7,575.72	8,093.91	Gluconic acid	27,340.48	9,755.07
L- (+)-Tartaric acid	9,887.26	3,747.24	Citrulline	15,858.53	6,403.99
Threose	4,745.33	2,057.25	Leucine	161,769.30	91,346.70
2,6,11-Tetramethyldode cane	4,646.88	5,615.51	Undecane	7,243.38	8,664.52
Hexaric acid	7,944.43	3,103.89	Dehydroascorbic acid	20,056.54	5,028.53
Maltotriose	241,442.67	25,939.47	Palatinitol	3,941.11	1,773.02
4-Hydroxypyridine	28,945.86	19,877.46	Coniferin	26,530.85	9,684.50
Fructose 6-phosphate	19,115.49	4,217.66	D-Erythronolactone	36,228.78	14,509.97
Aconitic acid	67,075.29	22,259.06	4-Hydroxybutyric acid	208,287.20	265,813.64
β-Lactose	21,937.76	11,885.15	6-Aminohexanoic acid	479,720.16	742,466.58
D- (+)-Maltose	117,478.84	39,174.51	Dodecane	9,365.52	11,680.50
D- (+)-Cellobiose	14,461.61	6,032.62	N-Acetylneuraminic acid	16,283.58	4,623.84
α-Lactose	20,175.84	6,926.45	Xylose	13,292.83	5,892.06
Allose	34,504.93	13,564.81	β-Alanine	3,175.39	15,394.74
Trehalose	180,426.96	59,069.43	Succinate semialdehyde	5,859.25	3,029.49
Xylitol	19,241.05	9,679.84	L-Tryptophan	60,569.49	30,432.71
Arabitol	12,679.74	5,407.20	Mesoerythritol	82,194.48	28,700.24
Ribitol	925,218.33	405,403.81	Galactinol	129,805.41	27,617.37
Histidine	4,709.37	1,823.34	Cadaverine	47,808.50	19,662.28
Citraconic acid	45,216.49	12,387.99	Butylbid(trimethylsilyl)Amine	4,440.75	13,789.95
Butyrolactam	72,513.93	50,989.49	Lactitol	132,084.70	54,457.17
1-Methylgalactose	56,806.73	158,960.73	Maltitol	1,135,045.12	409,779.08
Docosane	7,543.83	9,031.37	Fumaric acid	55,830.62	16,264.78
2-Aminoethanol	112,062.01	63,300.51	O-Acetyl-L-serine	3,735.88	26,503.20
Digalacturonic acid	290,344.06	146,215.87	N-Vinyl-2-pyrrolidone	16,694.94	23,152.51
Tridecane	10,067.05	12,225.34	Glutamic acid	356,882.36	111,872.23
Palmitic acid	556,734.77	576,993.56	Glucose	4,109,570.83	2,418,260.93
Lactulose	41,230.97	13,891.03	Galactose	2,057,643.43	959,066.14
Erythronic acid	7,656.44	3,115.16	Hexose	2,062,720.40	579,944.82
Threonic acid	101,526.44	38,983.22	5-Nonanone	60,347.28	77,719.74
Lactic acid	66,323.93	74,314.80	2-Hydroxy-5-methoxy-3-(10-penta decenyl)-para-benzoquinone	6,868.88	5,819.02
Lactobionic acid	352,346.11	147,986.50	Octanoic acid	4,994.66	5,880.80
3,4,5-Trimethoxybenzoic acid methyl ester	7,626.74	2,181.70	Citramalic acid	8,962.55	3,055.47
Octanol	54,973.42	73,264.17	Piperidone	13,647.31	3,989.31
Succinic acid	102,665.98	24,932.00	Pentadecane	31,286.65	40,381.92

Oxalacetic acid	12,338.05	5,266.83	Glycylglycine	12,226.01	5,469.00
Putrescine	20,335.56	4,619.93	D-Panose	9,711.63	3,374.34
α-Ketoglutaric acid	8,941.50	1,933.57	Glutamine	4,690.97	2,889.87

Table S5 A total of 158 metabolites detected in fibrous roots of *P. notoginseng* based on GC-MS analysis

Metabolite name	Peak height (Inoculated)	Peak height (Uninoculated)	Metabolite name	Peak height (Inoculated)	Peak height (Uninoculated)
(-)-Shikimic acid	36,605.02	32,430.67	Inositol	1,759,220.63	1,750,759.50
1,6-Anhydroglucose	21,165.70	16,635.44	Isocitric acid	5,217,214.63	4,985,618.38
1-Heptanpol	11,656.65	10,392.20	Isoleucine	84,076.19	101,460.05
1-Octene	11,346.79	14,739.77	Isothreonic acid	12,296.53	11,578.02
1-Sec-butyl-1-ethylhyorazine	158,526.20	189,940.21	L- (-)-Arabitol	26,516.78	19,328.43
2,4,7-Octanetrione	51,337.50	57,464.18	L- (+)-Tartaric acid	13,573.78	11,324.52
2,5-Dihydroxypyrazine	7,182.52	4,598.22	Lactic acid	101,460.01	87,163.48
2,6,11-Tetramethyldodecane	7,879.29	8,836.79	Lactobionic acid	64,184.20	37,058.03
2-Aminoethanol	379,857.68	215,217.76	Leucine	75,140.41	103,030.89
2-Deoxy-d-glucose	57,906.58	61,889.15	L-Gulcono-1,4-lactone	62,705.34	55,538.20
2-Deoxytetronic acid	14,272.92	12,673.78	Linoleic acid	7,503.98	5,315.24
2-Hydroxyglutaric acid	25,870.95	12,180.32	L-Ornithine	62,889.76	44,036.61
2-Isopropylpyrrolidine	243,781.18	306,694.91	L-Tryptophan	11,995.80	20,355.95
3-Hydroxy-3-methylglutarate	7,696.77	5,927.34	Lysine	67,441.85	62,121.76
3-phosphoglycerate	15,155.61	12,830.53	Lyxose	20,938.66	17,811.53
4,8-Dimethylquinoline	41,004.39	21,514.34	Malic acid	5,268,179.13	5,013,171.13
4-Aminobutyric acid	434,956.84	222,840.80	Malonic acid	72,026.31	111,147.34
4-Hydroxypyridine	19,908.34	21,975.87	Maltitol	206,507.95	75,064.89
4-Pentenyl isothiocyanate	16,341.71	19,489.11	Maltotriose	139,027.94	86,146.12
5,6-Dihydrouracil	651,613.14	530,497.23	Mannitol	46,389.67	34,748.13
5-Aminopentanoic acid	102,024.25	56,955.86	Mannonic acid	7,689.52	6,313.00
Aconitic acid	14,009.10	16,872.85	Mannose	34,518.55	21,362.42
A-Ketoglutaric acid	57,506.76	41,800.43	Melibiose	30,885.24	14,120.18
Alanine	23,529.71	13,305.01	Meso erythritol	9,232.44	10,271.88
α-Lactose	11,122.27	9,280.84	Myo-Inositol	12,017.05	10,873.52
Arabinose	14,422.29	11,953.02	Myristic acid	131,621.20	130,265.55
Arabitol	16,112.95	18,486.01	N-Acetyl galactosamine	73,390.57	70,598.67
Arbutin	7,688.05	7,186.16	N-Acetyl-d-glucosamine	62,788.49	47,079.38
Ascorbic acid	5,642.83	13,985.94	N-Acetylglutamate	2,788.00	11,209.20
Asparagine	19,890.61	49,320.22	N-α-Acetyl-l-ornithine	6,662.41	6,104.84
Asparagine dehydrated	21,420.88	12,549.67	N-Carbamoyl aspartate	14,811.25	11,488.39
Aspartate	88,337.24	248,505.83	Niacinamide	12,418.94	12,039.73
2-Aminobutanedioate	571,453.21	279,407.26	Nicotinic acid	129,791.95	120,981.80

β-Alanine	23,559.38	14,635.90	Nonadecane	26,663.61	26,648.58
Benzoic acid	19,083.49	21,779.44	Nonanoric acid	6,518.48	7,082.08
β-Lactose	15,838.46	12,776.14	N-Vinyl-2-pyrrolidone	21,005.78	26,358.30
Butylbis(trimethylsilyl)amine	6,323.78	8,825.96	Octanol	69,525.85	81,321.53
Butyrolactam	21,974.31	18,355.39	O-Phosphoethanolamine	17,723.80	22,288.79
Cadaverine	26,772.74	21,796.58	Oxalacetic acid	15,241.96	15,764.60
Citrulline	40,325.61	27,114.58	Oxalate	1,123,883.43	1,488,203.50
Conduritol-beta-epoxide	43,432.41	43,088.39	Oxamic acid	20,664.09	25,725.12
D-(-)-Mannitol	5,754.23	5,557.85	Oxoproline	2,299,828.00	2,105,168.88
D-(+)-Maltose	49,447.91	39,154.40	Palmitic acid	1,225,582.13	1,229,087.13
Decane	21,519.07	24,720.39	Panose	15,691.88	12,593.56
D-Glucose 6-phosphate	15,467.52	12,030.42	Pantothenate	16,331.72	14,768.71
D-Glucuronic acid	32,738.53	18,421.39	Pentadecane	59,909.16	65,643.81
Diethoxy methyl octylsilane	6,256.84	6,895.14	Phenylalanine	6,729.02	21,105.50
Dihydroorotic acid	312,930.76	399,741.79	Phenylethylamine	7,431.73	5,240.17
DL-2,3-Diaminopropionic acid	9,311.44	15,469.02	Phosphate	4,567,100.13	4,004,942.50
DL-β-Hydroxybutyric acid	14,294.73	5,578.12	Putrescine	73,936.18	9,228.17
Dodecane	13,544.12	15,525.37	Pyroglutamic acid	2,299,345.75	2,105,281.38
Fructose	1,172,829.00	1,015,361.85	Pyrophosphate meox	41,086.47	36,142.76
Fructose 6-phosphate	61,183.33	33,382.21	Quinic acid	19,672.21	13,342.27
Fumaric acid	118,248.10	107,348.30	Raffinose	8,371.77	5,323.88
Galactinol	92,352.40	60,349.21	Rhamnose	25,689.45	25,764.21
Galactitol	19,641.08	17,885.00	Ribitol	1,424,018.50	1,419,291.88
Galactonic acid	61,699.07	66,259.45	Ribose	10,500.11	11,361.42
Galactose	428,578.20	343,274.33	Serine	43,002.70	33,801.53
Gentiobiose	16,810.92	11,138.91	Sophorose	8,108.47	6,505.27
Glucarate	1,235,965.38	1,236,159.69	Sorbose	1,353,824.00	1,180,140.00
Glucono-1,5-lactone	7,756.09	7,921.77	Stearic acid	580,327.38	533,183.31
Glucose	1,822,150.50	1,516,048.88	Succinic acid	130,754.70	133,128.26
Glutamic acid	353,805.01	258,172.68	Sucrose	6,491,580.25	5,505,921.13
Glutamine	7,165.07	21,257.71	Sulfuric acid	22,084.53	5,704.27
Glutaric acid	12,203.84	8,972.48	Tagatose	12,075.48	13,148.50
Glyceric acid	52,874.99	59,350.06	Tetracosane	12,243.28	10,666.10
Glycerol	271,879.03	248,669.09	Tetrahydrolavandulol	21,760.10	24,874.96
Glycerol-3-galactoside	5,808.59	5,000.33	Threonine	30,352.18	24,263.31
Glycine	12,469.30	15,776.53	Trans-4-hydroxy-3-methoxycinnamate	8,532.70	8,756.23
Glycolic acid	31,810.91	26,974.04	Trehalose	112,199.33	73,770.65
Glycyl proline	69,792.41	64,724.20	Tridecane	17,044.53	19,179.87
Gly-Gly	21,942.95	24,896.62	Tyrosine	66,428.45	53,211.54
Heneicosane	11,535.77	11,289.84	Undecane	10,449.73	11,533.10
Heptadecane	36,081.97	38,111.29	Urea	486,343.50	497,175.46
Hexadecane	4,208.79	9,167.45	Valine	110,359.72	166,335.98
Hexaric acid	15,572.10	15,164.65	Xylitol	20,844.58	19,394.09

Hexose per	36,414.20	13,631.62	Xylonic acid	36,315.36	42,207.21
Hexose-6-phosphate	12,790.68	8,052.99	Xylonolactone	15,854.03	13,149.38
Hydroxycitronellal dimethyl acetal	8,046.91	9,021.38	6-Aminohexanoic Acid	802,773.88	853,905.31

Table S6 A total of 84 metabolites detected in root exudates of *P. notoginseng* based on GC-MS analysis

Metabolite name	Peak height (Inoculated)	Peak height (Uninoculated)	Metabolite name	Peak height (Inoculated)	Peak height (Uninoculated)
Glycolic acid	112,662.89	11,721.74	Nonadecane	13,425.15	11,429.03
Isoleucine	12,866.33	2,363.18	Heptanoic acid	6,304.92	2,894.52
2,4,7-Octanetrione	34,829.34	34,400.97	Serine	9,362.55	1,923.28
3-Hydroxypropionic acid	16,466.62	1,995.20	Oxalic acid	551,554.79	134,269.06
Threonine	12,470.45	11,927.08	Phosphate	33,394.17	6,255.30
Malic acid	13,217.89	2,417.91	Heptadecane	19,097.04	16,800.27
Sorbose	23,524.87	39,246.58	Oxoproline	92,315.18	9,386.81
Fructose	18,872.56	32,819.61	5,6-Dihydrouracil	3,859,659.23	700,200.29
Acetylsalicylic acid	5,370.80	440.30	Tyrosine	8,894.33	1,774.10
Inositol	12,271.54	3,863.66	Glycerol	1,761,312.74	172,642.41
L-Aspartic acid	14,474.66	291.89	Glucono-1,5-lactone	38,954.45	76,865.97
Aspartic acid	9,579.86	621.14	Tetracosane	6,914.25	5,468.93
Hypoxanthine	6,943.91	128.73	Ribose	247,713.41	12,580.74
Sucrose	9,993.34	16,003.30	3-Aminoisobutyric acid	6,016.51	5,988.22
4-Pentenyl isothiocyanate	10,816.28	11,486.35	Stearic acid	321,241.10	147,382.71
Glycine	8,864.77	8,766.92	Alanine	25,880.54	1,407.59
Decane	15,837.53	15,702.99	Ribonic acid	10,362.68	1,194.18
Uridine	4,638.33	1,160.41	Glyceric acid	71,070.11	5,071.31
Pinitol	7,943.01	80.06	Gluconic acid	15,322.70	5,896.67
Mannitol	3,794.21	5,974.38	Isohexonic acid	15,504.08	1,653.30
D- (-)-Mannitol	16,025.50	9,130.09	Leucine	16,413.54	3,560.97
L-Iditol	14,610.28	812.98	Thymine	13,557.01	1,132.45
Nonanoric acid	9,142.10	6,017.46	Palmitoleic acid	6,391.27	3,602.11
4-Hydroxypyridine	12,840.78	10,123.56	3-Deoxyhexitol	106,355.15	4,933.87
L- (-)-Arabitol	13,155.98	7,472.50	6-Aminohexanoic acid	549,368.53	584,999.09
Xylitol	9,594.35	24,330.74	Dodecane	9,459.85	9,383.98
Ribitol	582,990.30	107,778.27	Myristic acid	15,088.43	6,232.82
Docosane	13,225.60	10,547.04	β-Alanine	17,539.12	16,912.86
2-Aminoethanol	8,407.45	8,082.61	Butylbis(trimethylsilyl)amine	18,387.55	11,002.04
Tridecane	52,931.75	52,051.42	Dopamine	8,521.66	1,861.02
Plamitic acid	790,031.96	538,687.21	Fumaric acid	6,626.68	6,502.21
Uracil	11,512.57	608.35	DL-β-Hydroxybutyric acid	208,613.00	8,934.63
1-Sec-butyl-1-ethylhydrazine	110,001.78	115,017.40	N-Vinyl-2-pyrrolidone	15,065.53	15,792.94
Glutaric acid	9,642.02	1,013.29	Benzoic acid	22,897.28	9,928.64

Threonic acid	6,923.26	1,222.73	Glucose	5,809.06	7,896.39
N-Acetylornithine	33,073.00	30,272.67	5-Nonanone	60,251.52	59,829.63
Lactic acid	328,829.74	70,008.31	Octanoic acid	36,297.42	26,545.79
Octanol	53,375.89	52,681.84	Phthalic acid	9,317.81	442.43
Succinic acid	15,337.92	15,098.05	Urea	947,996.24	219,314.31
Oxalacetic acid	9,439.76	2,525.92	Xylonolactone	8,462.77	968.68
Putrescine	11,476.81	5,373.61	Pentadecane	40,895.85	37,145.85
Valine	29,318.71	4,778.97	Xylulose	14,024.83	1,790.92

Table S7 Significantly changed compounds (VIP>1, *p*<0.05) in root exudates with and without inoculation based on GC-MS or UPLC-Q-TOF-MS/MS analysis

Type	Class	Var ID (Primary ID Source)	Molecular formula	VIP	Fold change (Inoculated *vs.* Uninoculated.)	Probability	Peak height/area (Inoculated)	Peak height/area (Uninoculated)
GC-MS	Long-chain organic acids	Phthalic acid	$C_8H_6O_4$	1.23	36.27	0	11,705.80	322.75
		Palmitic acid	$C_{16}H_{32}O_2$	1.88	1.56	0.02	825,478.00	530,089.00
	Short-chain organic acids	Glutaric acid	$C_5H_8O_4$	1.08	15.01	0	12,029.90	801.37
		Glyceric acid	$C_3H_6O_4$	1.2	27.45	0	90,215.70	3,286.21
		DL-β-Hydroxybutyric acid	$C_4H_8O_3$	1.29	52.98	0	265,801.00	5,016.58
		Glycolic acid	$C_2H_4O_3$	1.12	17.06	0	139,614.00	8,183.00
		Succinic acid	$C_4H_6O_4$	1.1	1.04	0.03	15,622.90	14,977.60
		3-Hydroxypropionic acid	$C_3H_6O_3$	1.06	13.43	0	20,329.80	1,513.68
		Ribonic acid	$C_5H_{10}O_6$	1.01	11.07	0	12,999.90	1,174.60
		6-Aminohexanoic acid	$C_6H_{13}NO_2$	1.13	0.88	0.04	521,676.00	594,685.00
	Purines and pyrimidines	Hypoxanthine	$C_5H_4N_4O$	1.34	86.27	0	8,991.03	104.22
		Uracil	$C_4H_4N_2O_2$	1.22	32.7	0	14,757.00	451.24
		Thymine	$C_5H_6N_2O_2$	1.13	18.93	0	17,127.10	904.85
	Sugars	Ribose	$C_5H_{10}O_5$	1.22	34.09	0	317,680.00	9,318.14
		Fructose	$C_6H_{12}O_6$	1.17	0.64	0	16,612.40	25,765.10
		Sorbose	$C_6H_{12}O_6$	1.17	0.68	0	20,759.10	30,560.20
	Amino acids	L-Aspartic acid	$C_4H_7NO_4$	1.35	94.73	0	18,528.90	195.6
		Alanine	$C_3H_7NO_2$	1.22	43.51	0	32,941.00	757.08
		Oxoproline	$C_5H_7NO_3$	1.16	23.4	0	113,175.00	4,837.15
		Aspartic acid	$C_4H_7NO_4$	1.13	25.67	0	11,937.00	465.08
		Threonine	$C_4H_9NO_3$	1.1	1.08	0	12,721.80	11,812.20
		Glycine	$C_2H_5NO_2$	1.09	1.09	0.04	9,283.80	8,542.08
	Alkanes	Decane	$C_{10}H_{22}$	1.11	1.03	0.02	16,050.60	15,577.40
		Pentadecane	$C_{15}H_{32}$	1.09	1.13	0.01	41,701.90	36,869.20

		Heptadecane	$C_{17}H_{36}$	1.08	1.18	0	19,583.50	16,593.10
		Nonadecane	$C_{19}H_{40}$	1.07	1.22	0	13,877.50	11,407.30
		Tetracosane	$C_{24}H_{50}$	1.05	1.33	0	7,164.40	5,396.29
		Docosane	$C_{22}H_{46}$	1.04	1.34	0	13,921.80	10,397.90
	others	Acetylsalicylic acid	$C_9H_8O_4$	1.09	15.97	0	6,557.86	410.6
		Glucono-1,5-lactone	$C_6H_{10}O_6$	1.17	0.6	0.01	36,002.90	59,814.50
		1-Sec-butyl-1-ethylhydrazine	$C_6H_{16}N_2$	1.12	0.96	0.03	110,226.00	114,897.00
		2-Aminoethanol	C_2H_7NO	1.1	1.05	0.02	8,471.29	8,051.36
		4-Hydroxypyridine	C_5H_5NO	1.05	1.35	0	13,523.70	10,046.40
		Pinitol	$C_7H_{14}O_6$	1.4	144.84	0	10,281.10	70.98
		1-Deoxyhexitol	$C_6H_{14}O_5$	1.27	46.01	0	137,098.00	2,979.54
		Xylitol	$C_5H_{12}O_5$	1.24	0.38	0	6,588.41	17,526.70
		L-Iditol	$C_6H_{14}O_6$	1.22	33.17	0	18,791.40	566.45
		Glycerol	$C_3H_8O_3$	1.11	17.59	0	2,207,800.00	125,521.00
UPLC-Q-TOF-MS/MS	Amino acids	L-Serine	$C_3H_7NO_3$	2.1	1.55	0.03	12,241.48	7,907.08
	Sugars	D-Mannose	$C_6H_{12}O_6$	1.59	1.61	0.04	7,570.02	4,701.58
	Long-chain organic acids	Palmitic acid	$C_{16}H_{32}O_2$	1.88	2.21	0.02	31,041.73	14,015.99
		Azelaic acid	$C_9H_{16}O_4$	1.78	1.43	0.04	39,520.43	27,615.38
	Short-chain organic acids	2-Hydroxy-butanoic acid	$C_4H_8O_3$	2.17	0.64	0.01	15,153.05	23,584.13
		Propionic acid	$C_3H_6O_2$	1.97	2.06	0.03	36,752.38	17,837.27
		Dihydroxyfumarate	$C_4H_4O_6$	1.56	0.42	0.04	1,926.73	4,617.50
	Nucleotide and derivatives	Deoxyguanosine	$C_{10}H_{15}N_5O_5$	2.52	0.48	0	9,950.72	20,662.70
		Guanosine	$C_{10}H_{13}N_5O_5$	2.38	0.44	0.01	22,126.46	50,216.02
		Adenosine	$C_{10}H_{13}N_5O_4$	1.75	0.61	0.01	1,020,090.50	1,684,461.83
		Uridine	$C_9H_{12}N_2O_6$	1.41	0.67	0.03	266,620.75	398,000.94
		Cytosine	$C_4H_5N_3O$	1.51	0.57	0.01	29,714.95	51,959.56
	Others	Biocytin	$C_{16}H_{28}N_4O_4S$	2.7	2.43	0.02	968,814.55	399,046.66
		Glycerol	$C_3H_8O_3$	1.94	0.44	0.03	11,175.02	25,588.86
		4-Hydroxybutanoic acid lactone	$C_4H_6O_2$	1.87	0.36	0.02	40,851.01	114,710.95
		3-Chloro-L-tyrosine	$C_9H_{10}ClNO_3$	1.67	0.63	0.03	1,360.38	2,175.73
